# Nest boxes buffer the effects of climate on breeding performance in an African urban raptor

**DOI:** 10.1371/journal.pone.0234503

**Published:** 2020-06-24

**Authors:** Petra Sumasgutner, Andrew Jenkins, Arjun Amar, Res Altwegg

**Affiliations:** 1 FitzPatrick Institute of African Ornithology (FIAO), DST-NRF Centre of Excellence, University of Cape Town, Cape Town, South Africa; 2 Statistics in Ecology, Evolution and Conservation (SEEC), Department of Statistical Sciences, University of Cape Town, Cape Town, South Africa; 3 Konrad Lorenz Research Centre (KLF), Core Facility for Behaviour and Cognition, University of Vienna, Grünau/Almtal, Austria; INIBIOMA (Universidad Nacional del Comahue-CONICET), ARGENTINA

## Abstract

As the world’s human population increases, transformation of natural landscapes into urban habitats continues to increase. In Africa, rates of human population growth and urbanisation are among the highest in the world, but the impacts of these processes on the continent’s biodiversity remain largely unexplored. Furthermore, the effects of ongoing anthropogenic climate change are likely to be severe and to interact with urbanisation. Some organisms appear resilient to urbanisation, and even proliferate in human-modified environments. One such species is the peregrine falcon *Falco peregrinus* in Cape Town, South Africa. Using a long-term data set (1989–2014), we investigate the relationship between breeding attempts, timing of breeding and breeding performance under varying weather conditions. Exploring these issues along an urbanisation gradient, we focus on the role of artificially provided nest boxes, and their capacity to buffer against extreme weather events. Pairs in more urbanised areas, and particularly those in nest boxes, were more likely to breed and to commence breeding earlier. Additionally, pairs using nest boxes were more likely to breed in years with higher rainfall. Warm and dry weather conditions generally advanced the timing of breeding, although this relationship with weather was not seen for urban pairs using nest boxes. Furthermore, weather did not impact breeding performance directly (breeding success and fledged brood size), but timing of breeding did, with earlier breeders producing more fledglings. Our study shows that falcons breeding in specially provided nest boxes were less sensitive to local weather dynamics than pairs using more natural nest sites. This has important implications as it suggests that the managed provision of such nesting sites can help this key urban species to cope with extreme weather events, which are predicted to increase with climate change.

## Introduction

Globally, more people live in urban than in rural areas, and by 2050, 66% of the world’s population is projected to be urban-dwelling [1]. Rapid urbanization poses new challenges for species–some may go locally extinct, while others may adapt or relocate [2]. In addition to urbanisation, climate change is also putting considerable pressure on global biodiversity [3, 4]. The United Nations Secretariat considers the combination of urbanisation and climate change to be the most significant current source of global environmental change [5]. However, these two factors are unlikely to operate independently, and climate change is likely to exacerbate the negative effects of urbanisation on biodiversity. Indeed, a recent study on butterfly phenology suggests that their combined negative effect may be synergistic rather than simply additive [6].

The spatial and temporal distribution of most species is ultimately climate-driven [7], and urbanisation is well known to alter local climate, for example via the urban heat island effect [8–10]. The heat island effect mainly influences temperature and humidity, both key climatic variables controlling important life-history processes (e.g., productivity and survival). Climate change is thus likely to be felt particularly acutely in urban habitats, and heat waves are predicted to increase dramatically in intensity, duration and frequency in future urban environments [11]. Clearly, understanding how organisms respond to human-modified environments, and how their phenology and reproductive success are affected by varying weather conditions in these altered systems, is critical to developing effective measures to mitigate these effects.

Top predators are particularly vulnerable to anthropogenic disturbance because of their slower life histories [12] and are susceptible to human-wildlife conflict and associated direct persecution [13, 14]. Significant losses of apex predators through this processes can have cascading effects at the ecosystem level and contribute further to global biodiversity loss [15]. The presence of apex predators also has the potential to maintain or restore ecosystems and confer resilience against globally threatening processes, including climate change and biological invasions [16, 17]. Conversely, some apex predators are functionally and behaviourally plastic enough to exploit foraging opportunities created by urbanisation (e.g. [18–20]) and may even contribute to the loss of less adaptable prey species or competitors [21].

Today, Africa and Asia are urbanising faster than any other regions in the world, but comprehensive studies of the ecological effects of this phenomenon are generally lacking [22]. This is especially concerning in areas such as the Cape Peninsula, South Africa – a biodiversity hotspot located on the South-Western tip of the African continent – where the impacts of climate change are likely to be particularly severe [23–26] and where warming has been among the most rapid of all South African regions since the late 1980s [27, 28].

In this study, we focus on a peregrine falcon (*Falco peregrinus*, hereafter ‘peregrine’) population resident on the Cape Peninsula. This is a predatory, cliff-nesting species, with a pan-global distribution, which has colonised many large cities around the world [29–31]. Our study population includes pairs nesting on natural cliffs, and pairs nesting on buildings and quarries located in the suburban and urban environments of Cape Town. Some urban pairs nest in specially provided nest boxes, which facilitated the initial colonisation of this highly modified habitat [29]. The distribution of this population allows us to compare the performance of pairs breeding in natural situations with those using the full spectrum of urban habitats, and to examine the relative influence of weather conditions on these birds.

Many urban-breeding falcon species prefer sheltered locations on buildings, and particularly specifically provided nest boxes, over other nesting locations. They tend to exhibit higher territory occupation and earlier egg-laying at such sites [32–34], and sometimes higher productivity [35]. This appears to be a consistent phenomenon, which is known from several urban falcons [32, 35], and nest boxes are recognised as potentially useful tools to deploy in efforts to conserve threatened species [36]. Furthermore, urban birds might be affected differently by climate change than those in more natural habitats, which might be linked to a more predictable food resource within urban environments [37] or to artificial nest sites with a different micro-climate compared to natural nests [38]. In raptors, some species benefit from the accumulation of avian prey in urban areas [19, 30, 31] which might undergo lower fluctuations than natural prey populations. Another example are falcon species breeding in nest boxes which might be more sheltered against extreme weather events than conspecifics nesting in more open environments such as cliffs [39–41]. In peregrines, this positive effect of nest boxes has for example been seen in an arctic population [42]. The impact of weather events on peregrine breeding performance has furthermore been documented in several populations, including on the Cape Peninsula, which usually exhibit reduced breeding success when experiencing high rainfall while incubating in spring and early summer [42–49]. Additionally, in the Arctic, a study showed that the increased frequency of heavy rain explained the recent decline in peregrine breeding productivity, most likely in combination with ectoparasite outbreaks [49], and demonstrated experimentally that nestling survival can be improved by the provision of nest boxes [42].

We predict that within our study population: I) Breeding probability is correlated with urbanisation and the availability of nest boxes, with pairs occupying more urbanised territories and nest boxes, more likely to attempt to breed. II) Timing of breeding and breeding productivity are correlated with urbanisation and the availability of nest boxes, with pairs occupying more urbanised territories and nest boxes likely to lay eggs earlier in the season and have higher productivity. Both predictions are based on the known positive effect that nest boxes have had on the species population increase [29] and the known higher abundance of many favoured prey species in Cape Town [19]. Thus, overall, we expect territories in urban areas and with nest boxes to be the most successful. III) Warm and dry weather conditions have a positive effect on breeding phenology, irrespective of the site location along the urban gradient. However, we expect the correlation between weather and the probability of a breeding attempt as well as weather and reproductive output to be stronger at natural nest sites that are more exposed to the elements than at urban nest boxes. Thus, overall, we hypothesise that nest boxes offer more protection against extreme weather events than more open nest sites on buildings, natural cliffs and quarries, and could be a suitable tool for conservation in the face of global change.

## Material and methods

### Study area and field methods

Our study area extends over C. 1300 km^2^, from the Tygerberg Hills in the north (-33.772404°, 18.566170°) to Cape Point in the south (-34.359070°, 18.49749°), and includes most of the Cape Town metropole and Table Mountain National Park. The area has a Mediterranean climate, with locally variable winter rainfall (average about 800 mm, range 400–2000 mm per annum). Temperatures range from an average daily minimum of about 9°C in winter, to an average daily maximum of about 25°C in summer.

**Fig 1:**
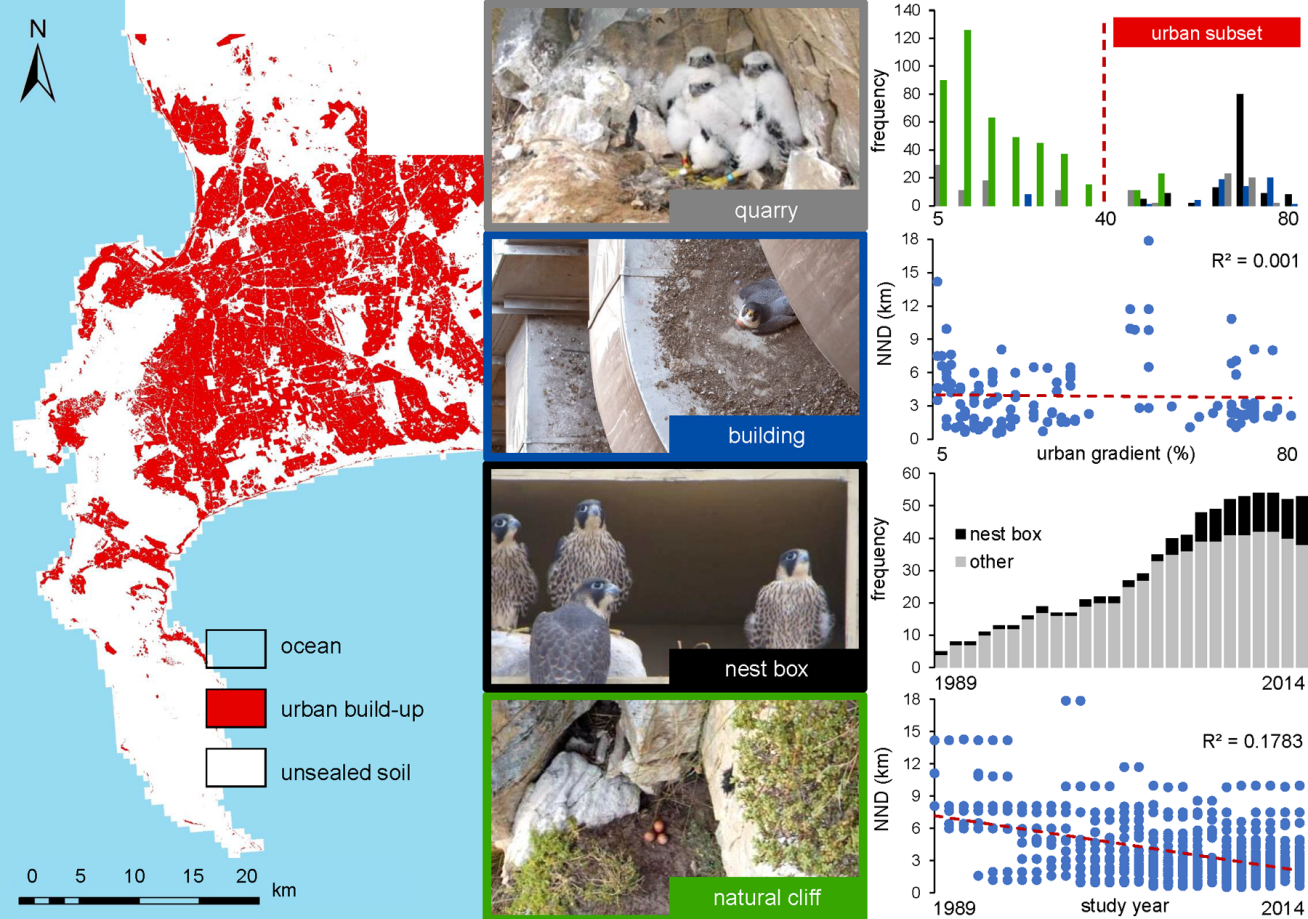
Study area and distribution of urban areas on the Cape Peninsula, South Africa. Peregrine falcon nest types were categorised as nest boxes (black), nests on buildings without boxes (blue), in quarries (grey) and on natural cliffs (green); photos: Andrew Jenkins. Note that nest locations are senstive information and were not plotted on the map to protect the species. Distribution of the Peregrine falcons and nearest neighbour distances (NND; n = 779 territory occupation records) in relation to **(top)** the urban gradient [% urban cover] and **(bottom)** the increasing population density over years [1989–2014]. Red dashed lines indicates the threshold for the ‘urban subset’, which included all four nest types but was restricted to nest locations with >40% urban cover, and the regression lines.

Peregrines in our study area breed on inland or marine cliffs, in stone quarries and on buildings [44] (Fig 1). We studied the timing of breeding and breeding performance in this population from 1989–2014. Initially, the available cliff habitat (>200 natural cliffs of varying size and aspect [50]) was searched regularly for resident pairs of birds. Thereafter, the known cliff-nesting population was monitored annually, and the remaining habitat was searched periodically for new territories. Over the first 10 years, surveys of birds in urban habitats were limited to the single known nest site on a building and a small number of disused stone quarries. As the presence of more urban pairs became apparent, searches were extended to include all the taller buildings in the southern two-thirds of the Greater Cape Town area, as well as all of the active and inactive stone quarries within the city limits (see details in [29]). Nest surveys and monitoring commenced from August 01 each year (laying generally occurs in late August or early September; [51]). Territories were checked either by observing falcon behaviours (conducted from a distance using 40x binoculars and a 20-40x spotting scope), or by directly accessing the nest site to determine nest contents. As far as possible, all previously occupied territories were visited at least once each year (in addition to any new territories found). The completeness of data collection varied between territories and years: some nest sites were inaccessible, some territories were particularly remote and were only visited once per season (to determine occupancy and breeding success), and time and capacity constraints meant that some territories were not surveyed annually (missing values). In contrast, the more accessible territories were visited at least three times per season spread over the breeding cycle, with multiple visits to the nest site itself in a subset of these. Since only territories found occupied during the first visit were monitored further throughout the season, we recorded all following parameters for occupied territories only: ‘breeding probability’ (binary: no breeding attempt ‘0’; or sign of incubation, eggs, nestlings or fledglings ‘1’), ‘breeding success’ (binary: failed ‘0’; or large or fledged young present ‘1’) and ‘fledged brood size’ (count data, 1–4 young fledged per territory). In the absence of information from later in the cycle, young surviving to >21 days old (ringing age) were considered to have fledged, thus, late brood failures might not have been fully captured.

‘Lay dates’ of successful nests were generated as Julian days by back-dating (subtracting 32 d + 2d per egg for the egg-laying and incubation period–e.g. [46]) from hatching dates, which in turn were calculated according to nestling age (gauged in terms of stage of plumage development, size and weight) at the time of inspection (e.g. [47]). If nestlings couldn’t be directly aged, we allocated ‘lay periods’ to breeding attempts, derived from the timing of behavioural changes associated with the onset or termination of incubation, or by direct observation of what subsequently proved to be incomplete clutches. This method allowed us to allocate the onset of breeding to one of six, two-week-long lay periods (1 = late August, 2 = early September, 3 = late September, 4 = early October, 5 = late October, 6 = early November).

### Nest boxes, the urban gradient and nearest neighbour distances

Levels of urban development were quantified within a radius of 3000 m around each nest site. This buffer size was based on the home range size (100% minimum convex polygon, average daily range of 20.2–25.9 km^2^) measured during the breeding season by radio tracking adults in adjacent territories [52]. This method has previously been used in other raptors to estimate the degree of urbanisation on a scale that is biologically meaningful for the studied species (e.g., [53–56]). Urban cover was calculated based on land cover classes from the 2013–2014 South African National land-cover dataset produced by GEOTERRA (Department of Environmental Affairs, 2015). This land cover dataset includes 72 land-cover types, mapped at a 30 m resolution, from which sealed, unproductive areas of land (buildings and traffic structures) were classified as “urban”. The percentage of urban cover was derived for each nest buffer using the packages *raster* [57], *sp* [58, 59] and *rgdal* [60] within the R environment. This variable represents our ‘urban gradient’ ranging from more natural territories with low urban cover to highly urbanised territories with high urban cover (Fig 1; up to 78%).

Peregrines nesting sites in the study area were allocated to one of four types: buildings with nest boxes (hereafter ‘nest boxes’), buildings without nest boxes (hereafter ‘buildings’), quarries and natural cliffs (Fig 1). Nest boxes were only installed on buildings. While nest boxes provide vertical and lateral protection from the elements in a standardised way, other nest types vary in the amount of shelter they provide. However, the availability of nest boxes was heavily confounded with the urban gradient (correlation coefficient ρ = 0.60), therefore, we considered two sets of analyses: dependent on the hypotheses tested we fitted either the full data set covering the full range of urbanisation (variable ‘urban gradient’) or we ran a subset of the data only considering urbanised pairs. These were in areas where both nest boxes and other nest types were present (urban gradient > 40% urban cover, see below), exploring the variable of ‘nest type’ in two levels: ‘nest box’ versus ‘other’ [pooling all other nest types together to achieve a more balanced data design; n = 126 breeding records in nest boxes versus 151 ‘other’ nests (buildings: n = 59; quarries: n = 58; natural cliffs: n = 34)]. The correlation between the urban gradient and nest boxes in this ‘urban subset’ was relatively low (ρ = 0.28); thus, we fitted both urbanisation and nest type as main effects and interaction terms in the subsequent models.

While there was some variation in the design of nest boxes used, most measured about 70 x 60 x 60 cm (width x height x depth), were open-fronted except for a 10 cm lip to contain a 5 cm layer of gravel and sand, and had perforated bases to allow rapid drainage of rainwater. Boxes were installed on buildings (1–3 per building), and were placed on high ledges, where human disturbance was limited. Nest boxes were placed to accommodate and manage falcon pairs that were already in residence, but where suitable nesting sites were not available. Nest boxes were first installed at a single site in 1989, and were subsequently installed at a further 14 sites from 1999 to 2013 (see [29] for more details, and Fig 1).

In our analyses, we also controlled for potential density effects as number of breeding pairs within the study area grew significantly over time [29]. We included nearest neighbour distance (NND, in meters, log transformed) as the distances to the closest present neighbour per year (see e.g. [61, 62] for recent literature on density dependence in other raptor populations). We chose NND because studies exploring different measurements found the distance to nearest neighbours to be the best predictor for density dependence (e.g. [63]).

### Weather data

Weather data for the Cape Peninsula were obtained from the South African Weather Service (station Cape Town Airport: -33.9630°, 18.6020°, altitude: 42 m, approximately 16 km from the centre of our study area). To characterise the weather experienced during the peregrine breeding season, mean (average), min and max temperature, total sum of rain, total number of rain days and rain intensity (sum of rain divided by rain days) over three periods, each of three months in length, assigned according to the peregrines’ annual breeding cycle: ‘courtship period’ (capturing the pre-breeding and early courtship period) during the winter (May 01 to July 31); ‘laying period’ (which includes the later courtship, incubation and early nestling stage; egg-laying starts in August) in spring (August 01 to October 31); and, ‘nestling period’ (which included the later nestling stage and fledging) in summer (November 01 to January 31). For each period, these variables were then reduced by principal component analysis (PCA) to account for correlations between temperature and rainfall measurements. We used PC1 and PC2 for each period in subsequent analyses. Low scores on PC1 courtship period (‘pc1court’: 38% of variance) represent wet weather; and low scores on PC2 (‘pc2court’: 26% of variance) cold weather. Low scores on PC1 laying period (‘pc1lay’: 51% of variance) represent cold and wet weather, high scores represent warm and dry weather; low scores on PC2 (‘pc2lay’: 28% of variance) represent warm temperatures, high scores represent cold weather. Finally, for the nestling period, low scores on PC1 (‘pc1nest’: 39% of variance) represent cold and dry conditions; and on PC2 (‘pc2nest’: 30% of variance) cold and wet conditions. (PCA scores in S1 Table in S1 File and relationship between raw weather data and PC1 and PC2 scores plotted for all three periods of the peregrine breeding cycle in S2 Fig in S1 File).

### Statistical analyses

#### Weather trends

To explore weather trends over the course of the study period we fitted generalized additive models (R-package ‘gam’ [64]) with annual sum of rain [mm] and average annual temperature [°C] respectively as response variables, and year as predictor (1989–2016) using a cubic regression spline. This was done to confirm that the Cape Peninsula does indeed experience strong weather trends as predicted by climate change [23, 24] which builds one foundation for our hypotheses and predictions regarding urban peregrines.

#### Breeding parameters

Timing of breeding (lay date) in relation to urbanisation was analysed with Linear Mixed Models (LMMs), while the corresponding analyses for ‘breeding probability’ and breeding performance were analysed with Generalized Linear Mixed Models (GLMMs).

To explore factors associated with breeding probability (binary data, modelled with a binomial distribution and a logit link function), we fitted urban gradient and the courtship period weather pc1court and pc2court as fixed effects, together with their interactions. Additionally, we fitted the nearest neighbour distance (NND) as a co-variate. In the urban subset-analysis (including nests with >40% urban cover), we also included ‘nest type’ (factor in 2 levels: ‘nest box’ versus ‘other’) and its interaction with pc1court and pc2court, and urban gradient. We removed non-significant interaction terms from the models to simplify the statistical approach and interpretation of the results.

The lay period (Gaussian distribution) was modelled as a response variable with the explanatory variables of urban gradient, the laying period weather pc1lay and pc2lay and the respective interactions between the urban gradient and these weather variables. We again controlled for any density dependence, fitting NND in the model. Additionally, we ran an urban subset-analyses (i.e. including only nest >40% urbanisation), in this analysis we also included ‘nest type’ and its interaction with pc1lay and pc2lay, and urban gradient. Where interaction terms were not significant, we fitted only the respective main effects. Lastly, we undertook a finer resolution analysis on lay date, which included specific lay date (to the nearest day) for successful nests (backdated from the age of the nestlings) as a response variable, but this was necessarily restricted to a smaller sample size of only those nests which successfully fledged young.

To analyse breeding performance, we used hurdle models [65] consisting of a binary part describing ‘breeding success’ (successful versus unsuccessful nests) and a zero-truncated Poisson part describing ‘fledged brood sizes’ (successful nests only with 1–4 chicks that fledged). Hurdle models had several advantages over alternative approaches since a) they account for the high number of zeros in the data set (when no young fledged); b) they account for the overdispersion in the count data (number of young that fledged); and c) the two parts of the model are biologically meaningful. We accounted for the timing of breeding (using lay period or lay date for breeding success and fledged brood size, respectively) in all models, using a logit link function for the binomial part of the model and a log link function for the count part. We fitted the interaction terms between the urban gradient and nestling period weather pc1nest and pc2nest, controlling for any density dependence by fitting NND. In the urban subset-analyses, we included ‘nest type’ and its interaction with pc1nest and pc2nest, and urban gradient. We removed non-significant interaction terms.

All models were fitted using R version 3.6.0 [66] with the packages *nlme* [67], *lme4* [68], *MASS* [69], *glmmADBM* [70] and *car* [71], and were visualised with *lattice* [72], *ggplots2* [73] and *effects* [74]. All quantitative variables were scaled (standardized to mean = 0 and standard deviation = 1) to bring the variables to comparable dimensions and to facilitate the correct interpretation of effect sizes for interaction terms. Residual distributions of the models were inspected visually to assess model fit. Throughout, we report model effect sizes (estimates ± SE, derived from the summary function in ‘lme4’); presented χ^2^ and p-values are based on an ANOVA Table of Deviance using Type II Wald χ^2^ tests for mixed models (Anova function in ‘car’ package). ‘Year’ and ‘territory ID’ were included as random terms to account for non-independence stemming from breeding data coming from the same territories over several years (territory ID), and multiple measures from different territories within the same year (year).

### Supporting analyses (results presented in supplementary material)

For breeding probability, we also explored an alternative model, to account for the fact that a newly found territory is by definition occupied and very likely actively breeding, which might create a confounding effect with urbanisation, since urbanised/nest box sites will have been occupied more recently and nest box sites were always known to researchers. To explore whether or not this was an issue, we fitted the ‘site type’ (factor in four levels: ‘natural cliff’, ‘quarry’, ‘building’ and ‘nest box’ over the full data set), the ‘year since the site was known’ (1989–2011 when the last new territory was found), the ‘site status’ (factor in 2 levels: ‘new’ and known ‘established’ sites) to account for these possible relationships, together with NND and the courtship period weather pc1court and pc2court. We did not consider the urban gradient in this alternative analysis, because it was heavily correlated with the predictors ‘year since site was known’ (ρ = 0.42) and with ‘site status’ (ρ = 0.60).

Additionally, we wanted to identify which weather period (courtship, laying, or nestling) had the greatest overall effect on eventual reproductive output. It was not possible to fit all pc axes at once, because of strong correlations between weather conditions during different time periods (e.g., pc2court was positively correlated with pc1nest, ρ = 0.61; pc2lay was negatively correlated with pc1nest, ρ = -0.51; and pc2court was negatively correlated with pc2lay, ρ = -0.36). Thus, we fitted 3 competing models for the response variables breeding success and fledged brood size respectively, with either the weather conditions during the courtship period, the laying period or the nestling period together with previously used co-variates and interaction terms, and compared the models using Akaike’s Information Criterion for small samples (AICc). We identified the ‘best models’ (model weight ω>0.50) for both data sets (i.e. full dataset, with the urban gradient as an explanatory variable; and the urban subset with nest type as an explanatory variable) and both response variables (breeding success and fledged brood size) and explored these best models further in a backward elimination process of interaction terms that did not feature significantly.

### Ethics statement

All individual colour ringing was conducted under a SAFRING license (1099 http://safring.adu.org.za/) and in strict accordance with current South African law. The monitoring was done with permission from CapeNature (permit number: 0037-AAA041-00059; https://www.capenature.co.za/), South African National Parks (http://www.sanparks.org/) and private land owners.

## Results

From 676 occupied territories between 1989 and 2014, we recorded 498 breeding attempts with an average lay date (of successful nests, n = 367) of 260 (egg-laying on 17^th^ of Sep) ± 14 SD and an average productivity of 1.72 fledged young (± 1.25 SD). There was no variation in nearest neighbour distances along the urbanisation gradient (Fig 1; average NND in non-urban areas = 3 710 m ± 2 655 SD (min 538 –max 14 288); and in urban habitats = 4 190 m ± 3 745 SD (min 1 095 –max 18 084)).

### Weather trends

Over the course of the study period, annual rainfall decreased (parametric effect F_(23,27)_ = 10.81, P = 0.003; non-parametric effect F_(23,27)_ = 2.46, P = 0.089); while average annual temperature increased (parametric effect F_(23,27)_ = 26.21, P<0.001; non-parametric effect F_(23,27)_ = 1.08, P = 0.376; estimated smoothing curves of cubic regression terms in S3 Fig in S1 File).

### Does weather or urbanisation influence breeding probability?

**Fig 2:**
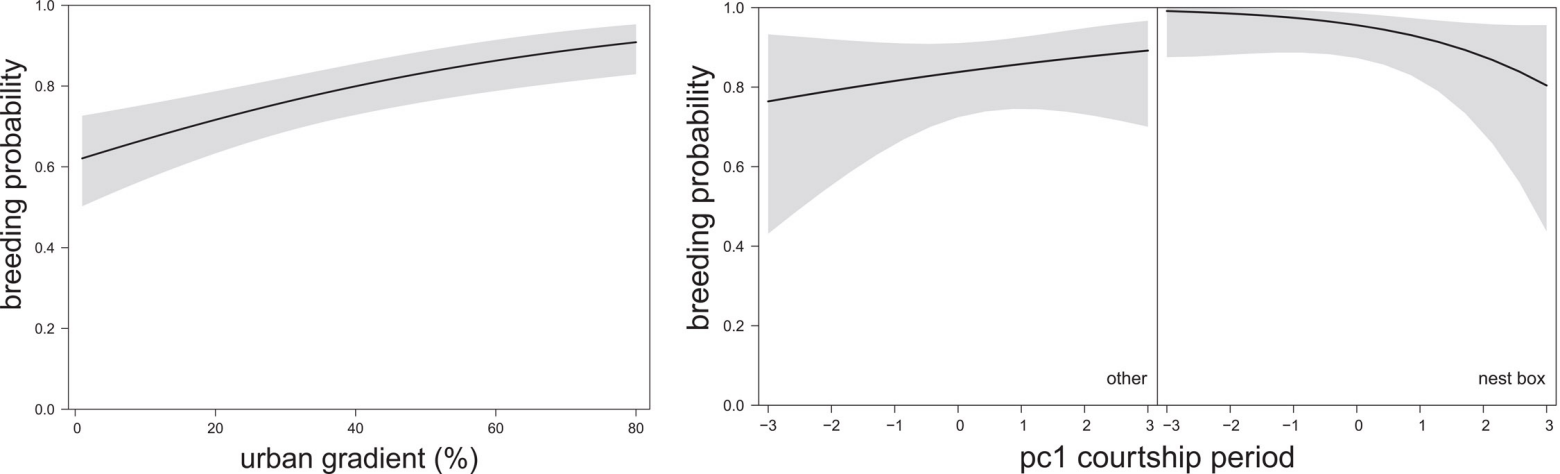
The relationship between breeding probability and **(a)** the urban gradient from the full data set (estimate 0.58 ± 0.15 SE, χ^2^_(n = 676)_ = 15.25, P < 0.001); and, **(b)** nest type (building with nest box versus other) in interaction with rainfall from the urban subset (including only nests with >40% urban cover; estimated interaction effect on the logit scale -0.71 ± 0.35 SE, χ^2^_(n = 255)_ = 4.00, P = 0.046) [negative values: high precipitation (total sum of rain, number of rain days and rain intensity); positive values: low precipitation] during the courtship period based on predicted values of GLMM, 95% CIs in shaded grey. Model details in Table 1.

Probability of breeding was influenced by the urban gradient, with more urbanised pairs more likely to initiate breeding than more rural/less urbanised pairs (Fig 2A). We found no indication of density dependence (NND) or of weather conditions during the courtship period influencing breeding attempts. The interaction between the urban gradient and weather was non-significant and was dropped from the final model presented in Table 1A.

**Table 1:** Generalized linear mixed effects model for breeding probability in relation to **(a)** the urban gradient (1–78% urban cover); and **(b)** nest type (nest boxes versus other), nearest neighbour distance (log transformed) and weather conditions during the courtship period (winter). Annual breeding attempts of 59 and 26 peregrine territories, respectively, over 26 years (1989–2014) on the Cape Peninsula. Significant estimates indicated in bold.

**(a) all nests n = 676 breeding attempts**	**estimate**	**SE**	**z-value**	**χ**^**2**^	**df**	**P-value**
*Intercept*	-0.01	1.49	-0.01			*0*.*993*
**Urban gradient**	**0.58**	**0.15**	**3.91**	**15.25**	**1**	**<0.001**
Nearest neighbour distance	0.33	0.42	0.80	0.63	1	0.426
Pc1 courtship period (rainfall)	0.09	0.10	0.87	0.76	1	0.384
Pc2 courtship period (temperature)	-0.01	0.12	-0.11	0.01	1	0.912
**(b) urban nests n = 255 breeding attempts**	**estimate**	**SE**	**z-value**	**χ**^**2**^	**df**	**P-value**
*Intercept*	0.41	3.46	0.12			*0*.*906*
Urban gradient	0.27	0.22	1.21	1.45	1	0.228
**Nest type (nest box) †**	**1.43**	**0.54**	**2.66**	**3.59**	**1**	**0.008**
Nearest neighbour distance	0.35	0.95	0.37	0.14	1	0.709
Pc1 courtship period (rainfall)	0.16	0.20	0.78	0.00	1	0.437
Pc2 courtship period (temperature)	-0.02	0.25	-0.10	0.01	1	0.925
**Pc1 courtship period (rainfall) x nest type †**	**-0.71**	**0.35**	**-2.00**	**4.00**	**1**	**0.046**

† Nest type ‘other’ were used as a reference category. Pc1: negative values: high precipitation (total sum of rain, number of rain days and rain intensity); positive values: low precipitation; Pc2: negative values: low temperatures (mean, min and max temperatures); positive values: high temperatures. See full matrix of factor loadings of PCA in S3 Fig in S1 File.

Using the urban subset, we found a significant positive main effect of nest type and a significant interaction between type and rainfall (pc1court) on breeding attempts. Pairs in nest boxes were more likely to breed in wet years (low values for pc1court) than pairs without nest boxes but the difference between nest types disappeared with decreasing rainfall (Fig 2B). From the supporting analyses, neither ‘year since the site was known’ nor ‘site status’ were significant (S4 Table in S1 File). The only significant association was essentially the same as our previous result, with pairs in nest boxes being significantly more likely to breed (S5 Fig in S1 File). Thus, to conclude it appeared that both urbanisation and weather appear to have an influence on breeding probability, but weather only appeared to influence pairs nesting in boxes.

### Does weather or urbanisation influence timing of breeding?

**Fig 3:**
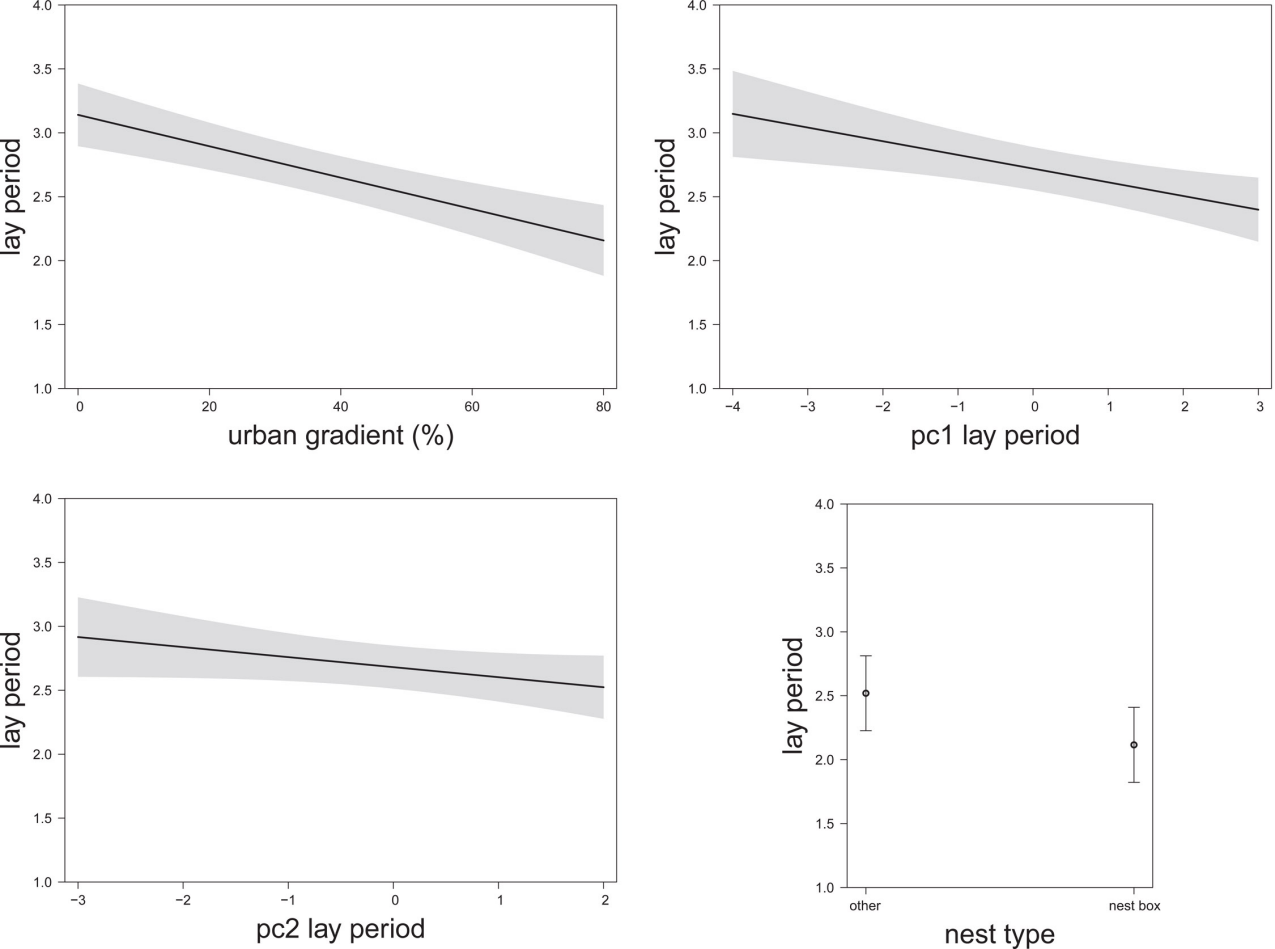
The relationship between the timing of breeding (lay period) and **(a)** the urban gradient (estimate -0.34 ± 0.07 SE, χ^2^_(n = 399)_ = 23.31, P < 0.001); **(b)** temperature and rainfall pc1 (estimate -0.11 ± 0.05 SE, χ^2^_(n = 188)_ = 5.69, P = 0.017) [negative values: cold and wet weather conditions; positive values: warm and dry weather conditions]; **(c)** temperature and rainfall pc2 (estimate -0.13 ± 0.06 SE, χ^2^_(n = 188)_ = 5.20, P = 0.023) [negative values: warm and wet weather conditions; positive values: cold and dry weather conditions]; and, **(d)** nest type (nest box versus other, urban subset with urban gradient >40% urban cover; estimate -0.41 ± 0.20 SE, χ^2^_(n = 188)_ = 4.33, P = 0.037) during the egg-laying period based on predicted values of LMM, 95% CIs in shaded grey. Model details in Table 2.

Peregrines bred earlier in more urbanised areas (Table 2A and Fig 3A) and when it was drier and warmer during the laying period (pc1lay; Table 2A and Fig 3B). There was also an indication (albeit marginally non-significant, estimate -0.08 ± 0.05 SE, P = 0.083) that breeding was earlier when it was drier and colder during the laying period (pc2lay; Table 2A and Fig 3C). This suggest that the main driver for the timing of breeding may be rainfall (i.e., dry weather) rather than temperature. We found no interaction between the urban gradient and weather during the egg-laying period.

**Table 2:** Linear mixed effects model for the timing of breeding (lay period) in relation to **(a)** the urban gradient (1–78% urban cover); and **(b)** nest type (nest boxes versus other), nearest neighbour distance (log transformed) and weather conditions during the egg-laying period (spring). Estimated lay period of 53 and 24 peregrine territories, respectively, over 26 years (1989–2014) on the Cape Peninsula.

**(a) all nests n = 399 lay period**	**estimate**	**SE**	**t-value**	**χ**^**2**^	**df**	**P-value**
*Intercept*	*0*.*28*	*0*.*74*	*0*.*37*	*0*.*14*	*1*	*0*.*708*
**Urban gradient**	**-0.34**	**0.07**	**-4.83**	**23.31**	**1**	**<0.001**
Nearest neighbour distance	-0.03	0.21	-0.13	0.02	1	0.895
**Pc1 lay period (cold/wet–warm/dry)**	**-0.11**	**0.04**	**-3.08**	**9.47**	**1**	**0.002**
Pc2 lay period (warm/wet–cold/dry)	-0.08	0.05	-1.74	3.01	1	0.083
**(b) urban nests n = 188 lay period**	**estimate**	**SE**	**z-value**	**χ**^**2**^	**df**	**P-value**
*Intercept*	*-1*.*61*	*1*.*19*	*-1*.*35*	*1*.*82*	*1*	*0*.*177*
Urban gradient	0.03	0.11	0.29	0.08	1	0.772
**Nest type (nest box) †**	**-0.41**	**0.20**	**-2.08**	**4.33**	**1**	**0.037**
Nearest neighbour distance	0.51	0.33	1.55	2.40	1	0.121
**Pc1 lay period (cold/wet–warm/dry)**	**-0.11**	**0.05**	**-2.39**	**5.69**	**1**	**0.017**
**Pc2 lay period (warm/wet–cold/dry)**	**-0.13**	**0.06**	**-2.28**	**5.20**	**1**	**0.023**

† Nest type ‘other’ were used as a reference category. Pc1: negative values: cold and wet weather conditions; positive values: warm and dry weather conditions; Pc2: negative values: warm and wet weather conditions; positive values: cold and dry weather conditions. See full matrix of factor loadings of PCA in supplementary material S3.

Finally, using the urban subset, we found pairs bred earlier in nest boxes than in other nest types (Table 2B and Fig 3D). From this analysis, the relationships with our weather variables was similar to the full data set (i.e., negative relationship with pc1lay and pc2lay, with both variables being significant; estimate -0.11 ± 0.05 SE, P = 0.017; and, estimate -0.13 ± 0.06 SE, P = 0.023, respectively).

**Fig 4:**
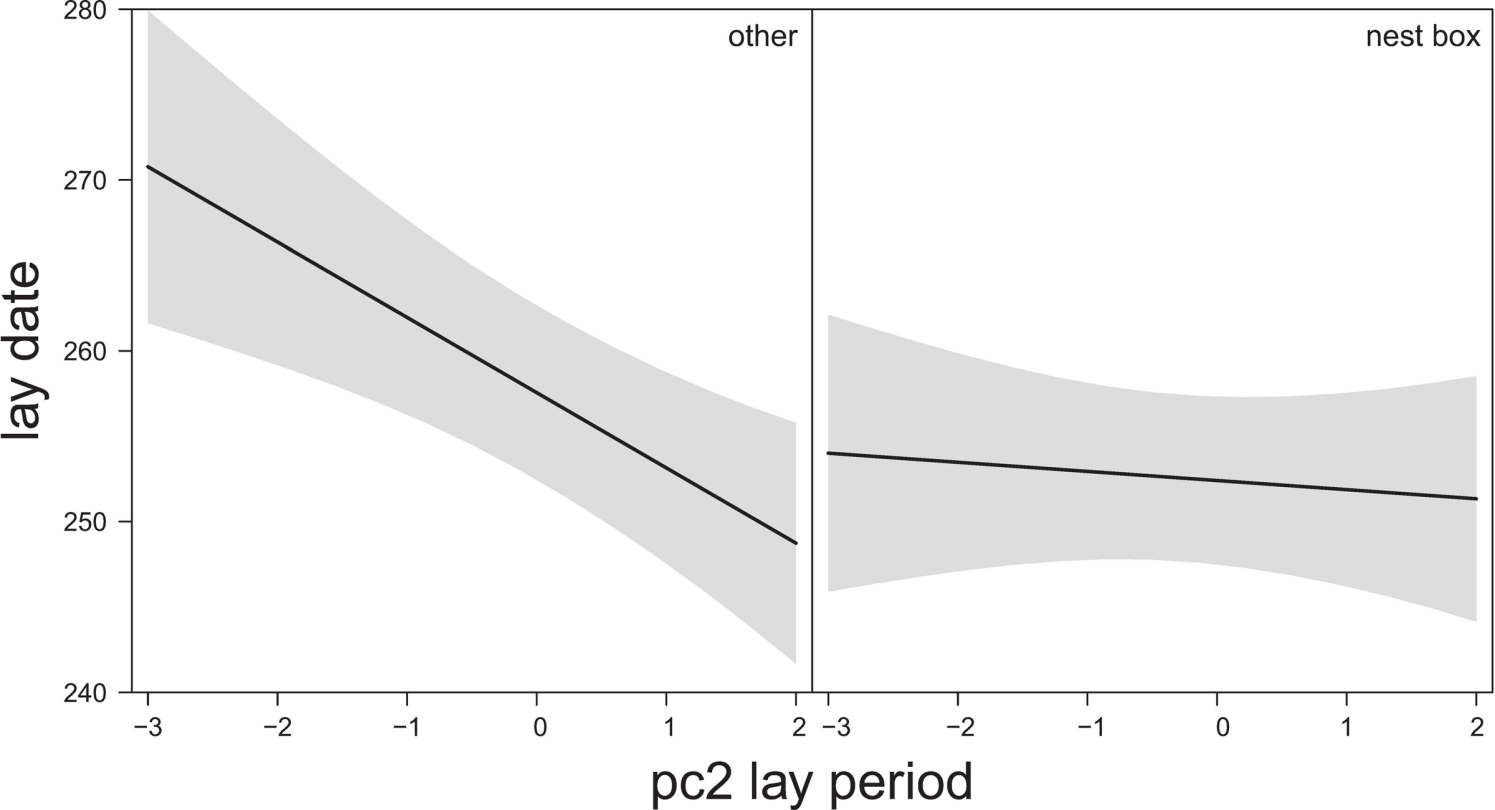
The relationship between the timing of breeding (lay date) for successful nests in the urban subset only and nest type (nest box versus other) in interaction with temperature and rainfall pc2 (estimate 0.28± 0.09 SE, χ2(n = 159) = 9.46, P = 0.002) [negative values: High average and high min temperatures with high precipitation (total sum of rain and rain intensity); positive values: Low temperatures and low precipitation (i.e., cold and dry)] during the egg-laying period based on predicted values of LMM, 95% CIs in shaded grey. Model details in Table 3.

We had more precise estimates of the egg-laying dates (i.e., to the nearest day, rather than a 2-week period) for successful nests, backdating lay date from the age of the nestlings. Results from this analysis were similar to those for the full data set considering the urban gradient as a key explanatory variable (Table 3A). However, for the urban subset, we found an interaction between nest type and weather (pc2lay), whereby there was no relationship between weather and laying for nest boxes, but for other nest types, laying occurred earlier in drier and colder conditions (Table 3B and Fig 4). This suggests earlier egg-laying under cold and dry weather conditions in other nest types was only apparent in successful nests. It was not seen in nest boxes or failed nests (independent of nest type).

**Table 3:** Linear mixed effects model for the timing of breeding (lay date) for successful nests only in relation to **(a)** the urban gradient (1–78% urban cover); and **(b)** nest type (nest boxes versus other), nearest neighbour distance (log transformed) and weather conditions during the egg-laying period (spring). Estimated lay period of 52 and 24 peregrine territories, respectively, over 26 years (1989–2014) on the Cape Peninsula.

**(a) all nests n = 316 lay date**	**estimate**	**SE**	**t-value**	**χ**^**2**^	**df**	**P-value**
*Intercept*	*0*.*62*	*0*.*80*	*0*.*78*	*0*.*60*	*1*	*0*.*439*
**Urban gradient**	**-0.36**	**0.08**	**-4.45**	**19.84**	**1**	**<0.001**
Nearest neighbour distance	-0.13	0.23	-0.59	0.35	1	0.555
**Pc1 lay period (cold/wet–warm/dry)**	**-0.13**	**0.04**	**-3.32**	**11.00**	**1**	**0.001**
**Pc2 lay period (warm/wet–cold/dry)**	**0.62**	**0.80**	**0.78**	**6.24**	**1**	**0.013**
**(b) urban nests n = 159 lay date**	**estimate**	**SE**	**t-value**	**χ**^**2**^	**df**	**P-value**
*Intercept*	-2.00	1.27	-1.58	2.48	1	0.115
Urban gradient	0.26	0.14	1.84	3.38	1	0.066
Nest type (nest box) †	-0.36	0.22	-1.63	2.64	1	0.104
Nearest neighbour distance	0.64	0.35	1.81	3.29	1	0.070
**Pc1 lay period (cold/wet–warm/dry)**	**-0.14**	**0.06**	**-2.48**	**6.15**	**1**	**0.013**
**Pc2 lay period (warm/wet–cold/dry)**	**-0.32**	**0.09**	**-3.61**	**13.00**	**1**	**<0.001**
Urban gradient x nest type †	-0.40	0.22	-1.78	3.16	1	0.075
**Pc2 lay period x nest type †**	**0.28**	**0.09**	**3.08**	**9.46**	**1**	**0.002**

† Nest type ‘other’ were used as a reference category. Pc1: negative values: cold and wet weather conditions; positive values: warm and dry weather conditions; Pc2: negative values: warm and wet weather conditions; positive values: cold and dry weather conditions. See full matrix of factor loadings of PCA in supplementary material S3.

### Does climate or urbanisation influence breeding performance?

**Fig 5:**
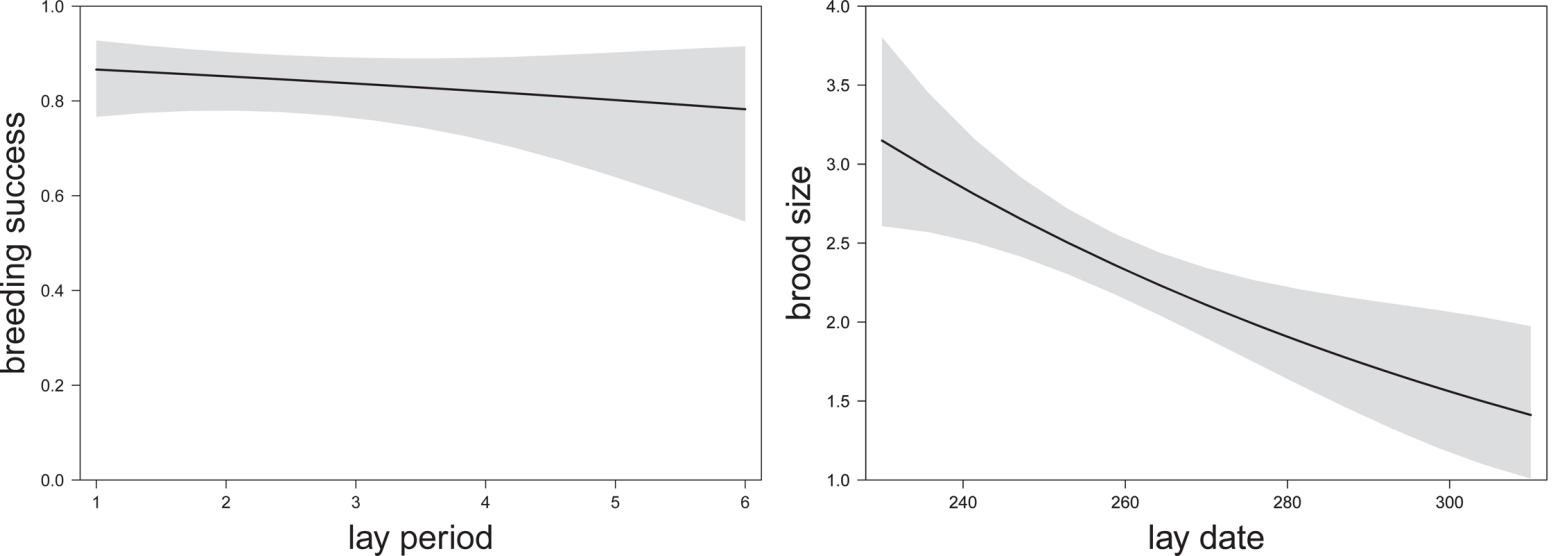
The relationship between (a) breeding success (binary part; estimate -0.11 ± 0.15 SE, χ^2^_(n = 399)_ = 0.55, P = 0.456); and, (b) fledged brood size (1–4 young, truncated part; estimate -0.19 ± 0.05 SE, χ^2^_(n = 316)_ = 13.70, P < 0.001) and the timing of breeding, based on predicted values of Hurdle model on the full data set, 95% CIs in shaded grey. Model details in Table 4.

Breeding success (binary–success/failure) was not associated with the urban gradient or weather variables, nor timing of breeding (2-week lay period, Table 4A, Fig 5A). However, earlier breeding did increase fledged brood size (based on backdated lay date, Table 4B and Fig 5B). Fledged brood size did not vary along the urban gradient, nor was it dependent on weather conditions during the nestling period. Thus, while timing of breeding did not influence the probability of nest success it was positively correlated with the number of chicks fledged.

**Table 4:** Hurdle model for breeding productivity **(a)** binary part: breeding success (n = 399); **(b)** truncated part: fledged brood size (n = 316 broods with a successful breeding outcome, 52 territories over 26 years); and, **(c/d)** n = 159/188 broods on the urban subset (including only nests with >40% urban cover).

**(a) Binary response: breeding success**	**estimate**	**SE**	**z-value**	**χ**^**2**^	**df**	**P-value**
*Intercept*	*2*.*85*	*1*.*91*	*1*.*49*			
Lay period	-0.11	0.15	-0.74	0.55	1	0.456
Urban gradient	0.21	0.16	1.31	1.71	1	0.191
Nearest neighbour distance	-0.34	0.53	-0.63	0.40	1	0.526
Pc1 nestling period (cold/dry–warm/wet)	-0.05	0.13	-0.39	0.15	1	0.695
Pc2 nestling period (cold/wet–warm/dry)	0.09	0.14	0.67	0.45	1	0.501
**(b) Truncated response: fledged brood size**	**estimate**	**SE**	**z-value**	**χ**^**2**^	**df**	**P-value**
*Intercept*	*0*.*74*	*0*.*53*	*1*.*39*			
**Lay date**	**-0.19**	**0.05**	**-3.70**	**13.70**	**1**	**<0.001**
Urban gradient	0.02	0.05	0.41	0.17	1	0.683
Nearest neighbour distance	-0.01	0.15	-0.09	0.01	1	0.931
Pc1 nestling period (cold/dry–warm/wet)	0.00	0.03	0.17	0.03	1	0.867
Pc2 nestling period (cold/wet–warm/dry)	0.00	0.03	0.02	0.00	1	0.980
**(c) Binary response: breeding success**	**estimate**	**SE**	**z-value**	**χ2**	**df**	**P-value**
*Intercept*	-2.39	4.36	-0.55			
Lay period	-0.13	0.28	-0.48	0.23	1	0.633
Urban gradient	-0.04	0.28	-0.13	0.02	1	0.898
**Nest box**	**2.10**	**0.75**	**2.80**	**5.49**	**1**	**0.019**
Nearest neighbour distance	1.08	1.24	0.88	0.77	1	0.380
Pc1 nestling period (cold/dry–warm/wet)	0.17	0.22	0.79	0.62	1	0.430
Pc2 nestling period (cold/wet–warm/dry)	-0.03	0.26	-0.10	0.21	1	0.645
Pc2 nestling period x nest type †	0.86	0.49	1.75	3.07	1	0.080
**(d) Truncated response: fledged brood size**	**estimate**	**SE**	**z-value**	**χ2**	**df**	**P-value**
*Intercept*	*0*.*48*	*0*.*88*	*0*.*54*			
**Lay date**	**-0.18**	**0.07**	**-2.64**	**6.98**	**1**	**0.008**
Urban gradient	0.03	0.07	0.36	0.13	1	0.721
Nest box	0.06	0.13	0.47	0.22	1	0.637
Nearest neighbour distance	0.08	0.25	0.30	0.09	1	0.760
Pc1 nestling period (cold/dry–warm/wet)	-0.01	0.04	-0.24	0.06	1	0.807
Pc2 nestling period (cold/wet–warm/dry)	-0.02	0.05	-0.29	0.09	1	0.769

† Nest type ‘other’ were used as a reference category. Pc1: negative values: low temperatures (mean and min temperatures); low precipitation (total sum of rain and rain intensity). Pc2: negative values: low temperatures (mean, min and max temperatures) and high precipitation positive (total sum of rain and rain intensity). See full matrix of factor loadings of PCA in supplementary material S3.

**Fig 6:**
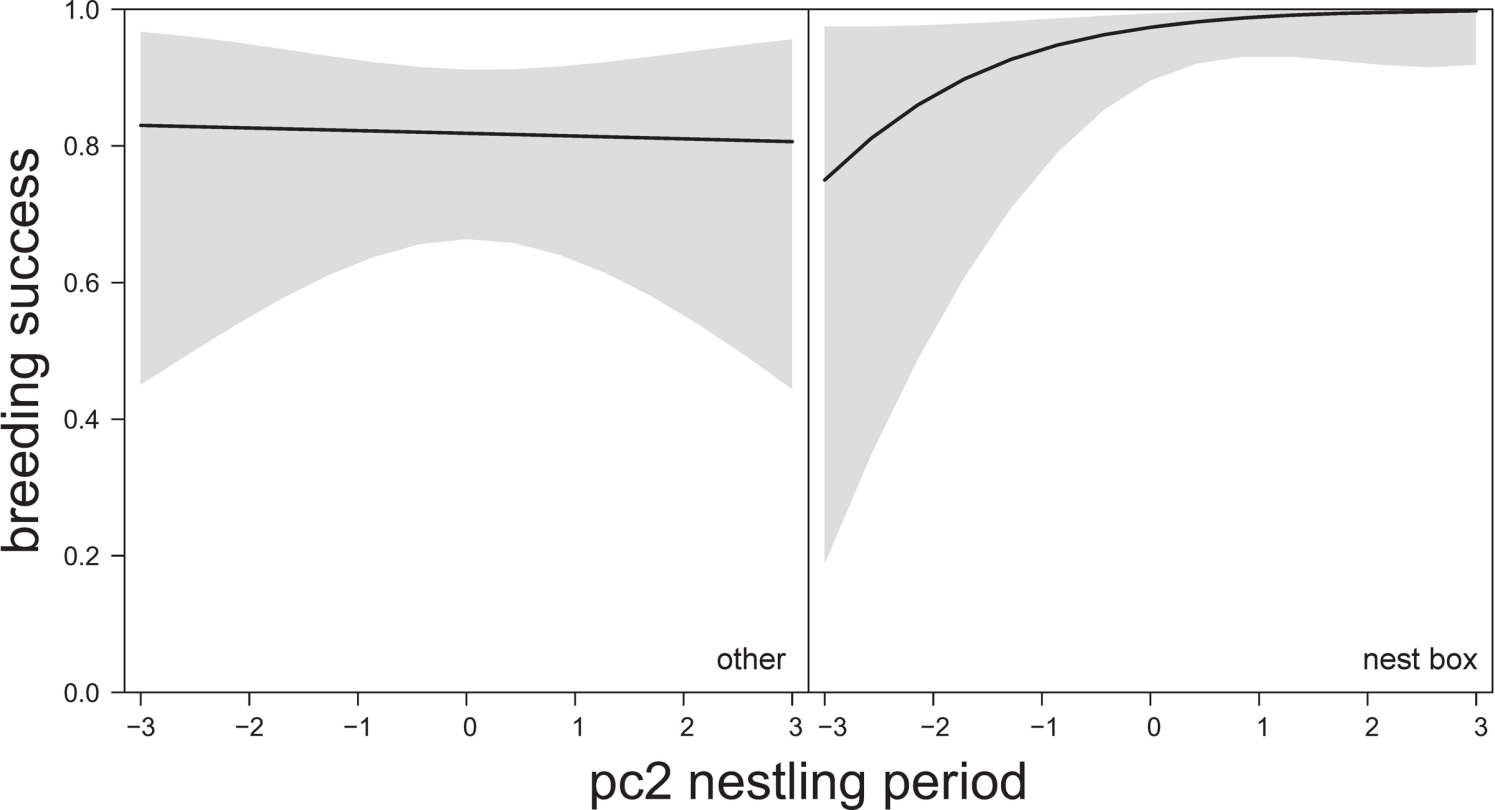
The relationship between breeding success and temperature and rainfall pc2 (estimate 0.86 ± 0.49 SE, χ2(n = 159) = 3.07, P = 0.080) [negative values: Low average, min and max temperatures and high precipitation (total sum of rain, number of rain days and rain intensity); positive values: High temperatures and low precipitation]; during the nestling period in the urban subset (including only nests with >40% urban cover) based on predicted values of Hurdle model (binary part), 95% CIs in shaded grey. Model details in Table 4C.

Using the urban subset, we found similar results. Timing of breeding did not influence breeding success (Table 4C), but did have an effect on fledged brood size (Table 4D). Nest box pairs had higher breeding success than pairs breeding in other nest types. There was also the suggestion of an interaction between nest type and weather variables during the nestling period (albeit marginally non-significant, estimate 0.86 ± 0.49 SE, P = 0.080), with a tendency for higher breeding success in nest boxes under drier and warmer conditions (pc2nest; Fig 6), but no such relationship for other nest types. Fledged brood size was independent of nest type and independent of weather conditions during the nestling period.

Finally, we compared models to explore within which time window (courtship, laying, nestling) weather conditions explained most of the variation in our two measures of breeding performance (breeding success and fledged brood size; S6 Table in S1 File model selection, S7 Table in S1 File best models). Variation in breeding success was most closely associated with weather conditions experienced during the egg-laying period (best model ω = 0.97; ‘pc2lay’ term estimate 0.40 ± 0.11 SE, P<0.001), and was highest when conditions were dry and cold (S8a Fig in S1 File). In other words, more nests failed when there was wet and warm weather experienced during the egg-laying period in spring (Aug-Oct). However, there was also a complex interaction term apparent (‘pc1lay’ additive term estimate 0.15 ± 0.09 SE, P = 0.034, ‘pc1lay × urban gradient’ interaction term estimate -0.14 ± 0.08 SE, P = 0.080) whereby breeding success was high, independent of weather conditions, for urban pairs. For rural pairs however, breeding success declined when it was wet and cold, whereas no such relationship was seen for more urban pairs (S8b Fig in S1 File).

We found that weather conditions during the courtship period explained the greatest variation in fledged brood size (best model ω = 0.71). There was a suggestion of an interaction between weather during this courtship period and urban gradient (‘pc2court × urban gradient’ estimate -0.07 ± 0.04 SE, P = 0.084), with a tendency for urban pairs to produce more young when it was colder during the courtship period (pc2court), whereas the opposite pattern was seen for less urbanised pairs, which tended to produce more young when it was warmer (S9 Fig in S1 File).

For the urban subset, no significant correlations were apparent when fitting nest type instead of the urban gradient. For the breeding success analysis, the best model was actually the one featuring weather during the nestling period (ω = 0.52) together with the previously identified suggestion of an interaction between pc2nest and nest type (Fig 6). The best model for fledged brood size was the one featuring weather during the courtship period (ω = 0.52), but none of the weather predictors were significant, with lay date being the only variable that apparently influenced fledged brood size.

## Discussion

**Fig 7:**
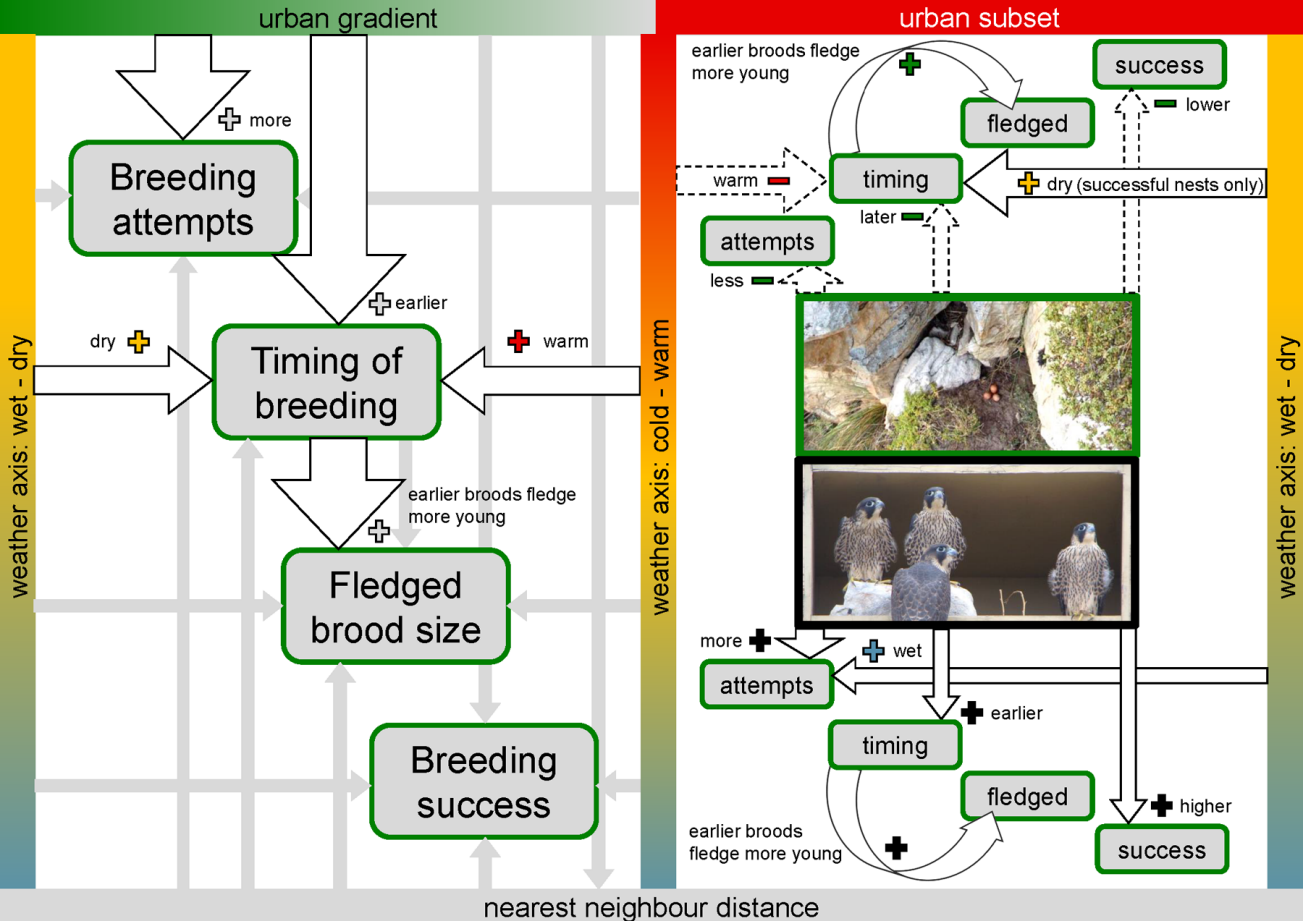
Visual summary. Breeding phenology and productivity of peregrine falcons on the Cape Peninsula. The relationships between variables analysed, their direction (“+” solid arrows: positive; and, “-”dashed arrows: negative) and their strength [black outline: significant effects (thick P<0.001; medium P<0.01, and narrow P<0.05); grey arrows: non-significant effects]. Displayed for four different parameters during the breeding cycle. (**left panel**) Relationships for the full extent of the urban gradient (0–80% sealed surface areas) and between all predictors considered. (**right panel**) Relationships for the urban subset (>40% urbanisation) where all nest types were available (“nest boxes” versus “other” nest types) showing statistically significant effects only.

Peregrine falcons at urban nest sites were more likely to breed and to breed earlier than pairs in less developed habitats. This was particularly so for urban pairs using sheltered nest boxes, which exhibited less variation in their breeding phenology in relation to local weather conditions compared to other pairs using more exposed nest types. Earlier breeding in urban areas also explained larger fledged brood sizes. Breeding success was independent of the timing of breeding, but broods in nest boxes were overall less likely to fail (Fig 7 Visual Summary).

### Earlier breeding in the city

Our study shows one mechanism by which nest boxes in urban areas could be more productive for peregrines: by shifting the breeding phenology toward early laying. Considerable research has shown that earlier breeding consistently leads to higher breeding performance: earlier clutches are larger, with higher hatching and fledging rates, and earlier broods are more likely to recruit into the breeding population [75–79]. Such correlations have also been recorded in other raptor species in Southern Africa [53, 79–81]. We found higher breeding probability and earlier egg-laying in more urbanised areas, and within these urban areas, higher breeding probability and earlier egg-laying by pairs using nest boxes on buildings. However, these results with the nest type should be interpreted with caution due to a more complex interaction with weather conditions (see discussion later). We offer two possible explanations. First, for specialised predators of birds, city centres can be a source of high prey abundance [30]. This has been quantified in other studies of urban raptors [82], including peregrines in the United Kingdom [31], where avian prey density was found to be higher in urban breeding territories than at rural sites; and Black Sparrowhawks (*Accipiter melanoleucus*) in Cape Town [19], where higher densities of many avian prey species were found in modified landscapes and urban areas than in more natural habitat types. In urban environments, the diet of peregrines typically consists of relatively few species, particularly pigeons and doves (*Columbidae*), and passerines (thrushes *Turdidae* and starlings *Sturnidae*; [83, 84]). These (sometimes alien) species proliferate in urban centres [19], presenting an unusually high abundance of prey that could enable female peregrines to reach breeding condition earlier in the year and promote earlier egg-laying [85].

Second, our results showed earlier egg-laying in this population under warm and dry weather conditions. One of the most consistent differences in the abiotic environment between urban areas and adjacent non-urban areas, is the urban heat island effect [8–10] with higher ambient temperatures and a lower fluctuation in diel temperatures in cities. This is caused by the heat-absorbing properties of urban structures (i.e., impervious surfaces and buildings) together with the scattering effects of air pollution, which traps radiated heat within the atmosphere of the city. The average temperature difference between cities and the surrounding countryside is usually around 2.9°C [86]. In our study we accessed detailed weather data for the entire length of the study period from one weather station and were thus not able to obtain finer scale data per territory which would be needed to shed light on possible local differences. However, we placed several weather stations at various locations in Cape Town between February and May 2019 (unpublished data from a different investigation), and found that the “urban” station in Observatory district was on average 2°C warmer than the “suburban” station at the University of Cape Town. Thus, it is likely that urban areas are overall warmer, and these warmer weather conditions could be sufficient to shift the timing of breeding by peregrine pairs towards earlier egg-laying.

### Timing of breeding and climate change

While we explored weather trends for the study period only, the observed reduction in annual rainfall together with the increase of average annual temperature are both in line with the long-term trends reported and projections for the Cape Peninsula (e.g., [24]). Phenological shifts are expected to occur due to climate change [87]. Given that the trend for drier and warmer weather is likely to continue, we would expect that the breeding phenology of the peregrine population will shift towards earlier egg-laying, and that this shift will be specifically pronounced in urban heat islands. However, we see in our results that within the urban subset dry weather only advances the timing of breeding in more natural sites, while egg-laying shows no variation in relation to weather in nest boxes. This indicates a buffering effect of nest boxes, which are also subject to higher breeding success. Therefore, in the future, more natural sites might catch up with nest boxes due to the overall warming climate.

Phenological shifts can further cause phenological mismatches by modifying vegetation growth [88] and altering food supplies of urban birdlife (including peregrine prey species). However, for peregrines there is no reason to believe that a phenological mismatch between predators and prey might occur due to climate change [89–91], as their main prey shows little annual variation in abundance [19].

### Micro-climate in nest boxes

We found pairs in more urbanised areas, and within urban territories, in nest boxes, were more likely to attempt to breed than pairs in more rural territories and in other nest types. We also found that within nest boxes, breeding was initiated more often when there was more rainfall. This indicates that peregrines may benefit from the greater protection from the elements that nest boxes offer specifically when the beginning of the breeding season was wet. Temperature did not seem to influence breeding attempts. However, enclosed nests, such as nest boxes, provide generally warmer and drier egg-laying conditions [38, 92]. A shifting breeding phenology often influences breeding performance positively. For example, Eurasian kestrels breeding in nests boxes and building cavities produced larger clutches compared to conspecifics occupying open-type nests [41, 93, 94]; this positive effect was assumed to be due to the favourable micro-climate that advanced egg-laying. In these kestrel studies, building cavities and nest boxes were available across a full range of urban, suburban and rural habitat types, allowing the authors to separate the positive effects of urban prey abundance and nest site protection in their interpretation of the results. This is not possible in our system, as nest boxes were confined to heavily urbanised areas where peregrines should also benefit from more abundant prey.

A study of peregrines in San Francisco that used micro-climate loggers at each nest site found that temperature and precipitation varied significantly between natural and anthropogenic sites [95]. Natural nest sites located on coastal cliffs were exposed to weather extremes including fog, which contributed to lowering temperatures, while nests on man-made structures in city centres experienced higher temperatures due to surface radiation and the urban heat island effect. A similar situation could be expected in our study system, and we did find indirect support for such a correlation: while warm and dry weather conditions overall advanced the timing of breeding in our population, this relationship was not apparent in nest box pairs where the timing of breeding was always earlier, independent of the measured average temperature and precipitation on the Cape Peninsula. Furthermore, it is known that heavy rainstorms negatively influence prey delivery rates [96] and increase nestling mortality in peregrines [42]. In fact, recent changes in rainfall patterns due to climate change could explain the long-term decline of peregrine annual productivity in the Canadian Arctic. In the same study, nest boxes were experimentally installed on cliffs, significantly increasing nestling survival by providing effective shelter from rainfall [42].

### Weather effects on reproductive performance

We found evidence for the importance of weather conditions during the pre-breeding and courtship period. This weather period proved to be most important in advancing the timing of breeding (as it was for urban kestrels [97]), but also in influencing fledged brood sizes (highest explanatory ability of models featuring weather conditions during the courtship period, as opposed to models considering weather during the nestling period itself). Breeding success, however, was mainly influenced by weather during the laying period, with dry conditions resulting in more successful nests. Similar effects have been noted for other peregrine populations, for example [45] and [48] both detected reduced breeding performance with higher rainfall during the early part of the breeding season. Our analysis also highlighted an interesting interaction between weather and the urban gradient, with a buffering effect again seen in more urbanised nest sites: while overall failure rates increased under cold and wet weather conditions during incubation, this correlation was not apparent in more urban sites, where breeding success was irrespective of temperature and rainfall. This result, albeit marginally not significant, could be important for urban conservation, and might become more relevant in the future given that extreme weather events will increase in frequency throughout South Africa [27].

Taken together, our results suggest that the timing of breeding matters more than varying weather conditions during later stages of the breeding cycle.

### Density dependence

We found no indication that any of the breeding related variables (breeding attempts, timing of breeding, fledged brood size and breeding success) were influenced by density of pairs present on territory (i.e. ‘nearest neighbour distance’ never featured significantly in any model). In our population, the most breeding attempts and the highest productivity (through the discussed shifts in breeding phenology whereby urban pairs lay their eggs earlier; and earlier pairs fledge larger broods) were recorded in urban areas where breeding pairs were supposedly most dense due to the installation of nest boxes [29]. Additionally to a higher nest site availability, smaller home ranges are reported for many urban raptor species which are, like the peregrine, avian prey specialists (e.g. [98–100]). A smaller home range size suggests sufficient prey availability, and thus less space is required to meet the dietary needs during chick rearing [101, 102]. However, differences in nearest neighbour distances were not very pronounced in our population. Both average NNDs, for non-urban areas (3.7 km) and for urban areas (>40% sealed soil, 4.2 km), are within the lower range reported for peregrines (for example in Great Britain, NNDs vary from 2.1–9 km [103]). This could be due to the vast abundance of suitable nesting cliffs on the Cape Peninsula, which supports an equally high territory density as urban sites.

### Future directions and urban conservation

Cities can feature diverse nesting opportunities for breeding raptors, including relatively natural structures (e.g., trees, quarries and sometimes even cliffs), and especially human-made structures (e.g., buildings). In this context, the provision of nest boxes can be useful in assisting populations to establish and grow [29]. Urban raptors that use human-made structures for nesting, are often negatively affected by widespread building renovations that specifically affect cavity nesters (e.g. [82]) and interventions meant to discourage the use of buildings by nuisance bird species prevalent in cities (e.g. spikes, or nettings at the façade [38]). Beside the benefit of nest boxes outlined in this study, nest boxes can play a very important role to compensate for such losses. Additionally, one would require the permission of the building owner to attach nest boxes, which ultimately ensures that artificial nest sites are only provided where the target bird is welcome to breed. This fact in itself might result in an overestimation of the importance of nest boxes (in our study the response variable ‘breeding attempt’), because nest boxes were only provided where breeding pairs were already present (which triggered the desire to provide a nest site) and where a positive attitude towards the falcons already existed. This reduces the amount of disturbance and brood loss that can occur when building owners are not appreciative towards birds breeding on their property [104]. Finally, one aspect that is still receiving little attention, is ecosystem services provided by raptors [105]. Anecdotal evidence suggests that ‘pest’ control services provided by peregrines nesting on buildings reduce maintenance costs relating to corrosive bird excrement [104]. Additionally, raptors influence prey populations by their very presence (i.e., establishing a ‘landscape of fear’ [106]) and thus have indirect effects that could be more important than their direct effects on prey species [107], including those considered ‘pests’ in cities [108]. In fact, in our study population, some nest boxes were installed in an attempt to attract peregrines and possibly achieve pre-emptive, eco-friendly control of local pigeon populations (AJ pers. comm.).

When taken together, the positive effect of nest boxes stemming from more breeding attempts (an indication that peregrines prefer this nest type), the fact that nest boxes can only be installed with permission of the building owner, which requires a positive attitude towards urban raptors, and the buffering effect that nest boxes have against varying weather events (this study) including heavy rainfall [42], suggest the importance of nest boxes for urban conservation.

## Conclusions

Key aspects for the successful establishment of an urban raptor population are the availability of nest sites and prey [109]. A similarly comprehensive study on the breeding performance of peregrines across urban and rural landscapes [31] suggested that higher prey density in urban areas was the main driver of higher productivity (in terms of breeding success, the number of young fledged in successful nests, and overall productivity including nest failures) in these habitats. However, information detailing breeding phenology–which proved to be a key mechanism for higher productivity in peregrines in our work–was not incorporated in their study. Together, these recent studies shed light on the success of avian predators in cities, and the complex interplay of prey abundance and nest site features, that might allow urban breeding pairs to breed earlier, which then translates into improved productivity. Our results also highlight the potential importance that nest box provision may play, for this charismatic urban adapted species, in coping with climate change in an urban environment.

## Supporting information

S1 File (PDF)
