# Supplementary material for: Nest boxes buffer the effects of climate on breeding performance in an African urban raptor
Source: PLoS One. 2020 Jun 24;15(6):e0234503. doi: 10.1371/journal.pone.0234503 (PMC7313736; doi:10.1371/journal.pone.0234503)

Nest boxes buffer the effects of climate on breeding performance in an African urban raptor

Petra Sumasgutner, Andrew Jenkins, Arjun Amar and Res Altwegg

Supporting Information S1 – Matrix of factor loadings of Principal Component Analysis (PCA).

PC axes used in statistical analyses and presented in the results are highlighted in grey.

**winter = COURTSHIP PERIOD**	**pc1court**	**pc2court**	**pc3**	**pc4**	**pc5**	**pc6**
Average temperature [°C]	0.03	**0.72**	0.26	0.16	-0.62	-0.01
Min temperature	-0.22	**0.68**	-0.26	-0.07	0.65	0.00
Max temperature	-0.02	0.04	**0.72**	-0.67	0.17	-0.01
Sum of rain [mm]	**-0.65**	-0.09	0.13	0.12	-0.06	0.73
Number of rain days	**-0.44**	-0.01	-0.48	-0.60	-0.39	-0.24
Rain intensity (sum/nb of rain days)	**-0.58**	-0.11	0.31	0.37	0.08	-0.64
**Importance of components:**						
Standard deviation	1.50	1.26	1.17	0.79	0.39	0.05
Cumulative Proportion of Variance	0.38	**0.64**	0.87	0.97	1.00	1.00
**spring = LAY PERIOD**	**pc1lay**	**pc2lay**	**pc3**	**pc4**	**pc5**	**pc6**
Average temperature [°C]	**0.51**	**-0.32**	0.02	0.02	-0.79	-0.10
Min temperature	**0.36**	**-0.50**	0.29	-0.60	0.41	0.07
Max temperature	**0.51**	**-0.13**	0.13	0.74	0.39	0.08
Sum of rain [mm]	**-0.42**	**-0.52**	0.04	0.19	-0.14	0.71
Number of rain days	**-0.34**	**-0.11**	**0.83**	0.17	-0.10	-0.39
Rain intensity (sum/nb of rain days)	**-0.26**	**-0.59**	-0.47	0.14	0.14	-0.57
**Importance of components:**						
Standard deviation	1.74	1.30	0.95	0.53	0.27	0.07
Cumulative Proportion of Variance	0.51	0.79	0.94	0.99	1.00	1.00
**summer = NESTLING PERIOD**	**pc1nest**	**pc2nest**	**pc3**	**pc4**	**pc5**	**pc6**
Average temperature [°C]	**0.45**	0.44	-0.23	-0.31	-0.67	-0.05
Min temperature	**0.47**	0.18	-0.61	0.08	0.61	0.04
Max temperature	0.07	**0.51**	0.56	-0.50	0.42	0.00
Sum of rain [mm]	**0.51**	**-0.42**	0.28	-0.08	-0.04	0.70
Number of rain days	-0.06	**-0.53**	-0.25	-0.77	0.07	-0.25
Rain intensity (sum/nb of rain days)	**0.55**	**-0.26**	0.35	0.23	0.02	-0.67
**Importance of components:**						
Standard deviation	1.52	1.35	1.00	0.85	0.35	0.12
Cumulative Proportion of Variance	0.39	0.69	0.86	0.98	1.00	1.00

Supporting Information S2 – Relationship between raw weather data and PCA scores for the courtship period, the laying period and the nesting period.

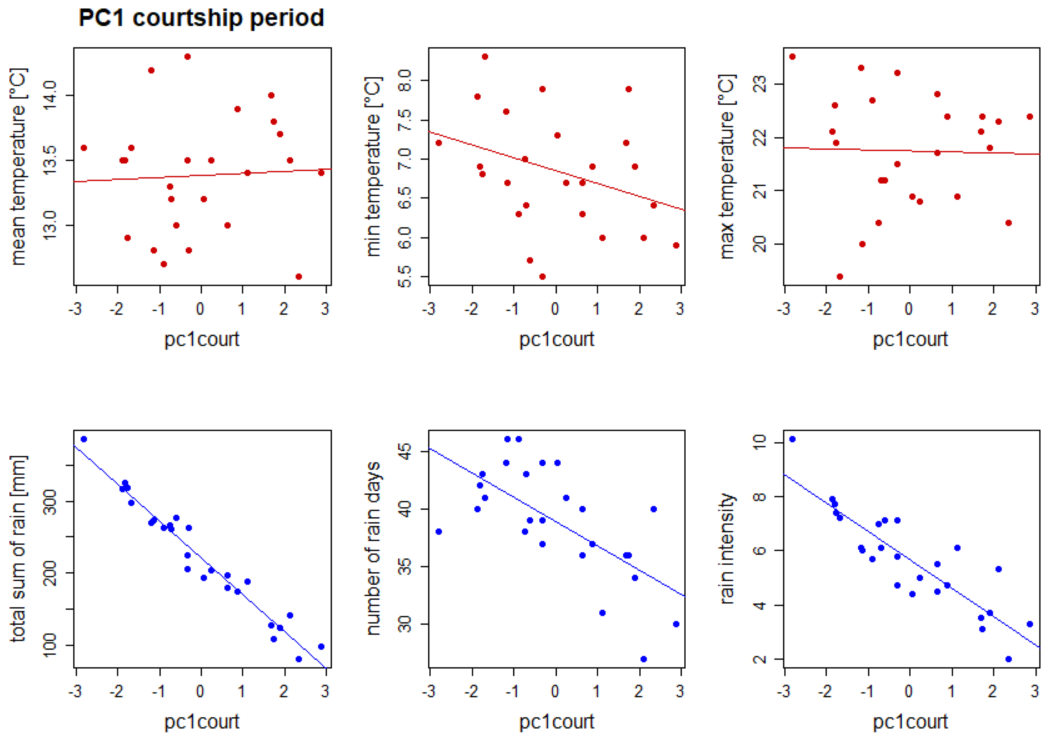

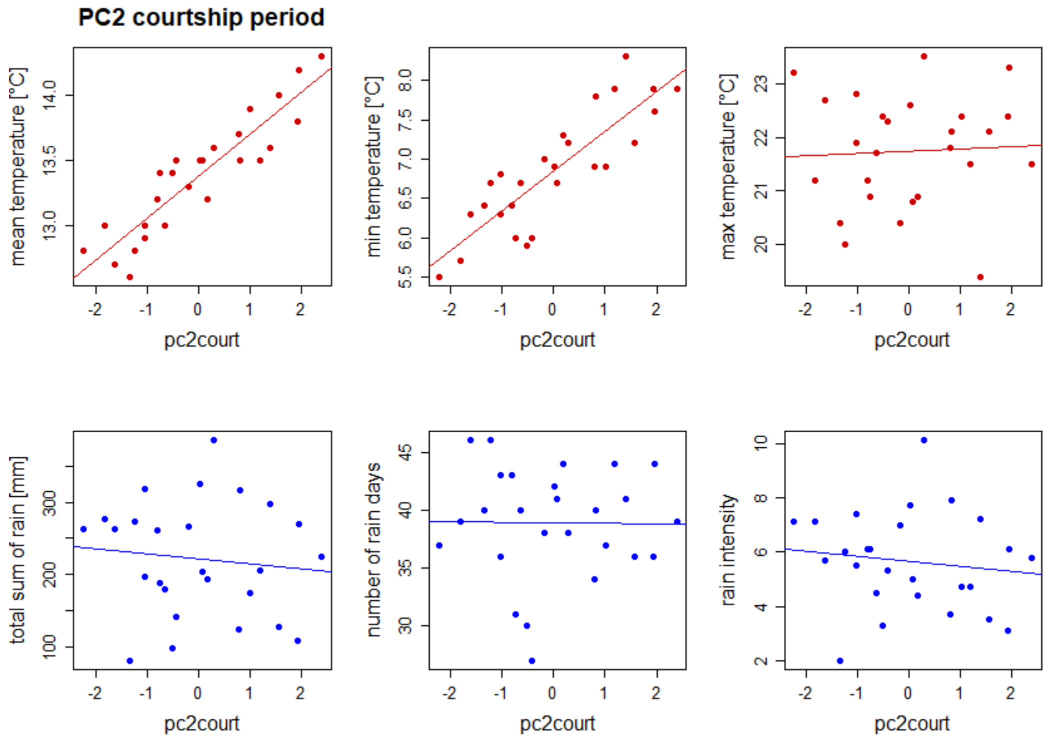

PC1 laying period

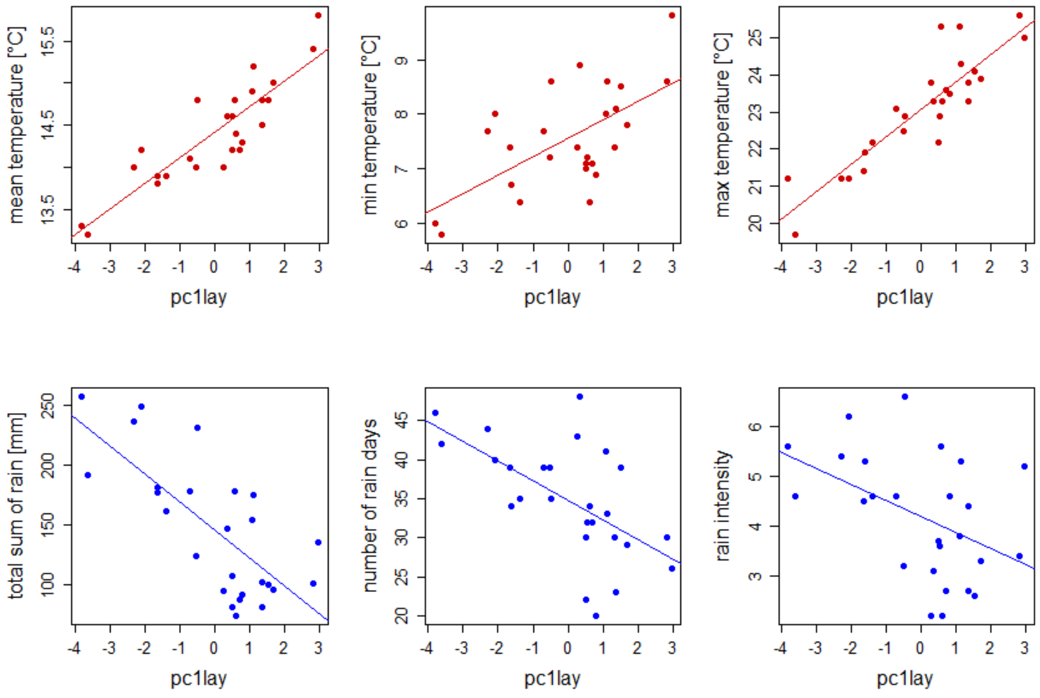

PC2 laying period

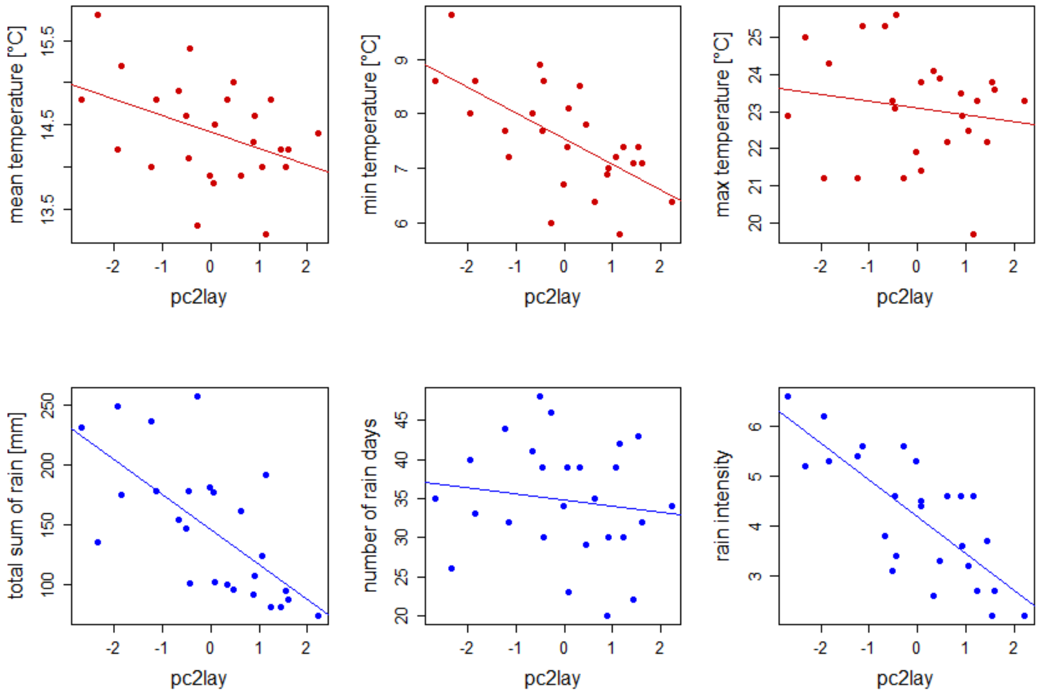

PC1 nestling period

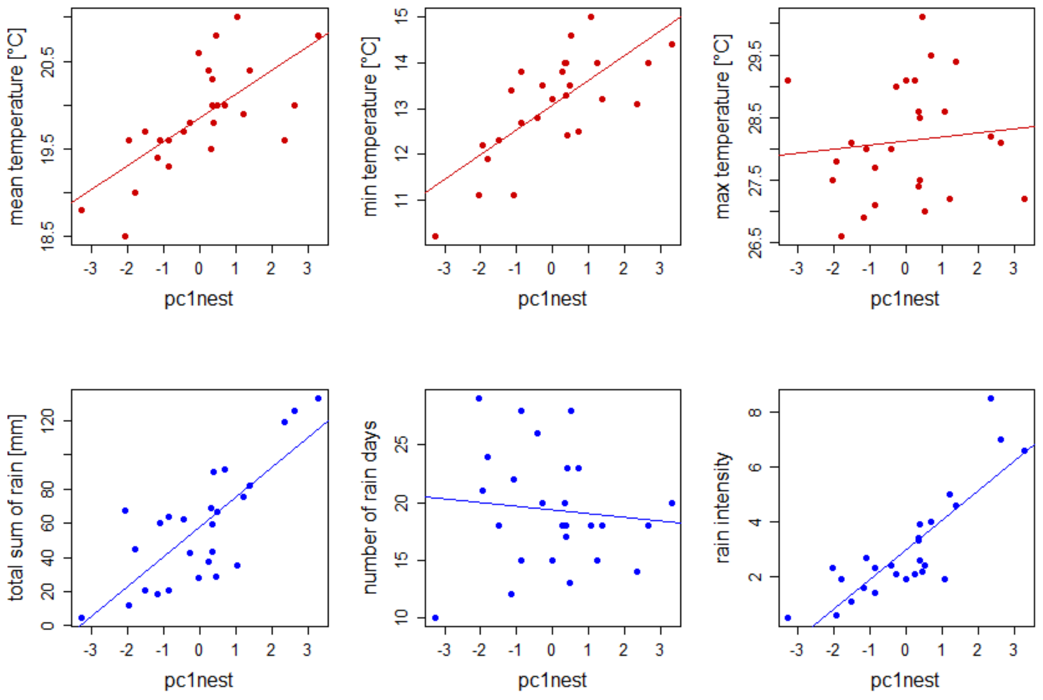

PC2 nestling period

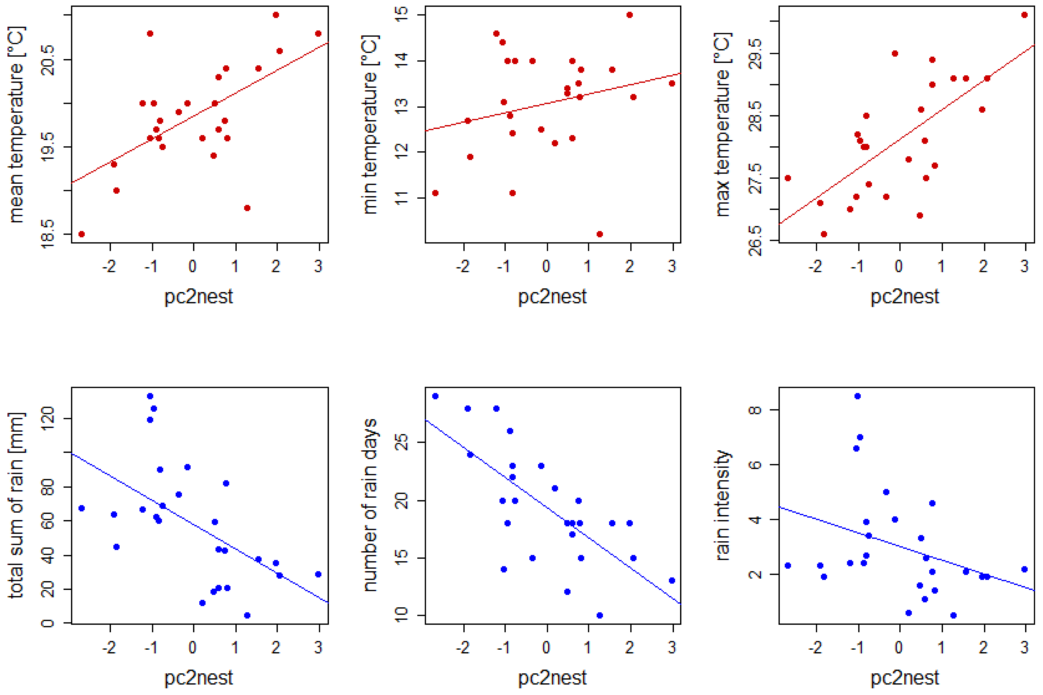

Supporting Information S3 – Estimated smoothing curve (cubic regression spline) and point-wise 95% confidence bands for sum of rain [mm] and average temperature [°C] over the course of the study period (from 1989).

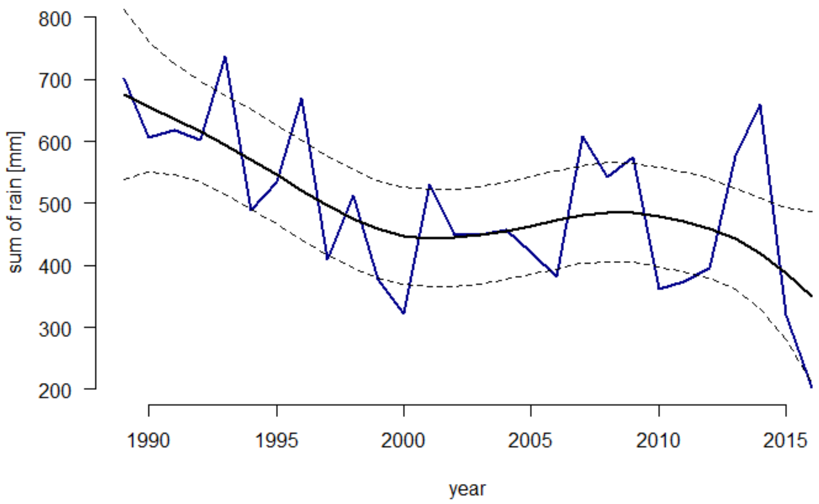

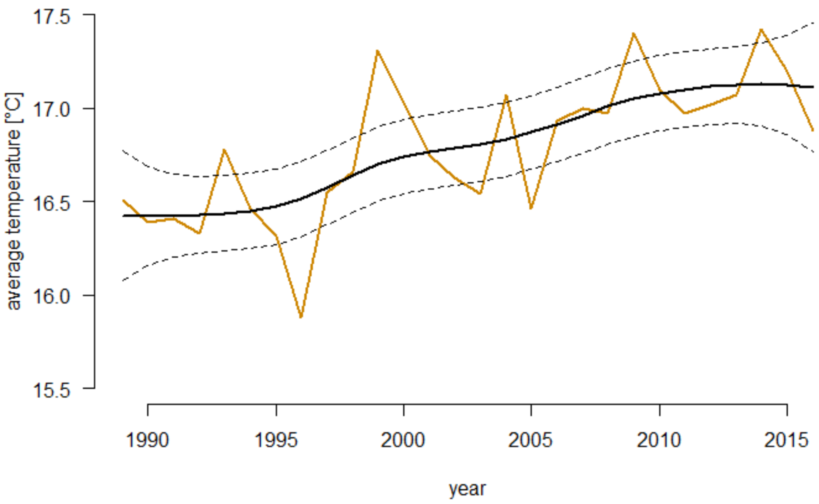

Supporting Information S4 – Generalized linear mixed effects model for the probability of breeding attempts in relation to site type (natural cliff, quarry, building or nest box), the year when the site was established (1989 until last new site in 2011) and site status (factor with two levels: new or established). n = 496 annual occupations of 37 peregrine territories with known site history.

**Response: Breeding attempt**	**estimate**	**SE**	**z-value**	**χ^2**	**df**	**P-value**
Intercept	3.08	1.85	1.66			
Site type‡ 'natural cliff'	-0.30	0.62	-0.47			
Site type‡ 'nest box'	1.92	0.71	2.71	12.81	3	0.005
Site type‡ 'quarry'	0.38	0.72	0.53			
Site known since [year]	-0.17	0.24	-0.70	0.49	1	0.482
Site status† 'new'	0.77	0.51	1.50	2.24	1	0.135
Nearest neighbour distance	-0.72	0.50	-1.43	2.06	1	0.151
Pc1 courtship period	0.05	0.08	0.69	0.48	1	0.488
Pc2 courtship period	-0.10	0.10	-1.01	1.02	1	0.313

‡ Site type: 'building' and † site status: 'established' were used as reference category.

Supporting Information S5 – The relationship between probability of a breeding attempt and nest type (building, natural cliff, nest box or quarry) based on predicted values of GLMM, 95 % CIs in shaded grey. Model details in Table S4.

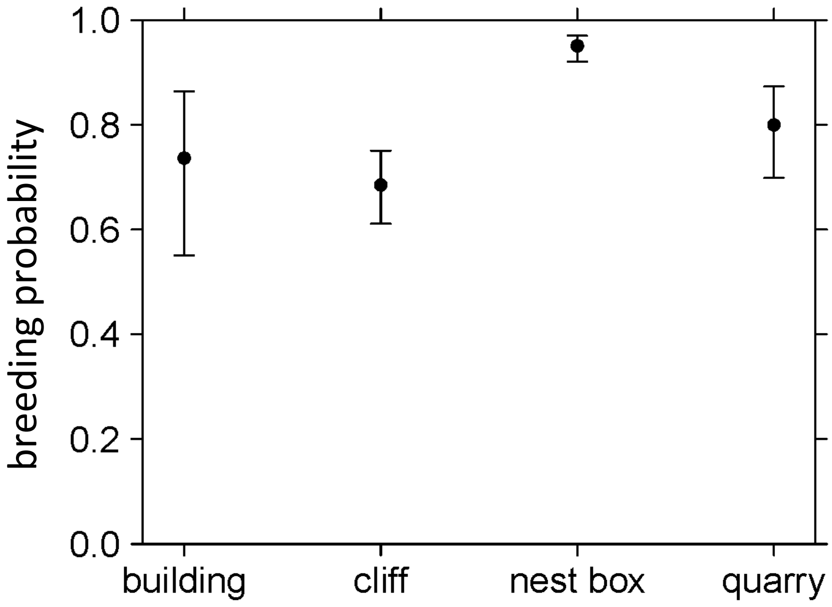

Supplementary Material S6 – Competing models for the response variables (a) breeding success; and, (b) fledged brood size with either the weather conditions during the courtship period, the laying period or and the nestling period together with previously used co-variates and interaction terms, compared by Akaike's Information Criterion for small samples (AICc) against each other.

**(a) model: response breeding success, full data set n=399**	**Df**	**LogLik**	**AICc**	**ΔAICc**	**weight**
lay period + pc1lay × ug + pc2lay × ug + pc1lay + pc2lay + ug + nnd	10	-174.79	370.2	0.00	0.97
lay period + pc1court × ug + pc2court × ug + pc1court + pc2court + ug + nnd	10	-178.42	377.4	7.26	0.03
lay period + pc1nest × ug + pc2nest × ug + pc1nest + pc2nest + ug + nnd	10	-180.92	382.4	12.26	0.00
**(b) model: response fledged brood size, full data set n=316**	**Df**	**LogLik**	**AICc**	**ΔAICc**	**weight**
lay date + pc1court × ug + pc2court × ug + pc1court + pc2court + ug + nnd	11	-348.11	719.1	0.00	0.71
lay date + pc1lay × ug + pc2lay × ug + pc1lay + pc2lay + ug + nnd	11	-349.48	721.8	2.74	0.18
lay date + pc1nest × ug + pc2nest × ug + pc1nest + pc2nest + ug + nnd	11	-349.99	722.9	3.77	0.11
**(c) model: response breeding success, urban subset n=188**	**Df**	**LogLik**	**AICc**	**ΔAICc**	**weight**
lay period + nb × ug + pc1nest × nb + pc2nest × nb + pc1nest + pc2nest + nb + nnd	12	-63.76	153.3	0.00	0.52
lay period + nb × ug + pc1court × nb + pc2court × nb + pc1court + pc2court + nb + nnd	12	-64.40	154.6	1.28	0.27
lay period + nb × ug + pc1lay × nb + pc2lay × nb + pc1lay + pc2lay + nb + nnd	12	-64.65	155.1	1.78	0.21
**(d) model: response fledged brood size, urban subset n=159**	**Df**	**LogLik**	**AICc**	**ΔAICc**	**weight**
lay date + nb × ug + pc1court × nb + pc2court × nb + pc1court + pc2court + nb + nnd	13	-182.96	394.4	0.00	0.52
lay date + nb × ug + pc1lay × nb + pc2lay × nb + pc1lay + pc2lay + nb + nnd	13	-183.69	395.9	1.46	0.25
lay date + nb × ug + pc1nest × nb + pc2nest × nb + pc1nest + pc2nest + nb + nnd	13	-183.78	396.1	1.64	0.23

ug = urban gradient, nnd = nearest neighbour distance (log transformed), nb = nest box, × = indicating an interaction term, Df = degree of freedom.

Supplementary Material S7 – Parameter estimates from the best hurdle models **(a)** binary part: breeding success vs egg-laying period climate (n=399); **(b)** truncated part: fledged brood size Vs courtship period (n=316 broods with a successful breeding outcome, 52 territories over 26 years); and, **(c)** truncated part on the urban subset of the data: fledged brood size Vs courtship period; n=159, only considering urbanisation scores ≥ 40% urban cover. Note that breeding success for urban nests only is not shown, as best model was identical to the model presented in **Table 4c**.

**(a) Binary response: breeding success**	**estimate**	**SE**	**z-value**	**χ^2**	**df**	**P-value**
Intercept	*3.45*	*1.85*	*1.86*			
Lay period	-0.11	0.15	-0.75	0.56	1	0.454
Urban gradient	0.30	0.17	1.76	3.42	1	0.064
Nearest neighbour distance	-0.50	0.52	-0.97	0.94	1	0.333
**Pc1 lay period (cold/wet – warm/dry)**	**0.15**	**0.09**	**1.73**	**4.51**	**1**	**0.034**
**Pc2 lay period (warm/wet – cold/dry)**	**0.40**	**0.11**	**3.78**	**14.30**	**1**	**<0.001**
Pc1 lay period × urban gradient	-0.14	0.08	-1.75	3.07	1	0.080
**(b) Truncated response: fledged brood size**	**estimate**	**SE**	**z-value**	**χ^2**	**df**	**P-value**
Intercept	*0.87*	*0.52*	*1.67*			
**Lay date**	**-0.18**	**0.05**	**-3.65**	**13.36**	**1**	**<0.001**
Urban gradient	0.04	0.05	0.85	0.49	1	0.485
Nearest neighbour distance	-0.05	0.15	-0.34	0.06	1	0.802
Pc1 courtship period (rainfall)	0.02	0.03	0.70	0.76	1	0.384
Pc2 courtship period (temperature)	-0.01	0.04	-0.16	0.12	1	0.732
Pc2 courtship period × urban gradient	-0.07	0.04	-1.73	2.99	1	0.084
**(c) Truncated response: fledged brood size**	**estimate**	**SE**	**z-value**	**χ2**	**df**	**P-value**
Intercept	*0.78*	*0.87*	*0.90*			
**Lay date**	**-0.17**	**0.07**	**-2.58**	**6.64**	**1**	**0.010**
Urban gradient	0.01	0.07	0.15	0.02	1	0.882
Nest box	0.07	0.13	0.59	0.34	1	0.558
Nearest neighbour distance	-0.01	0.24	-0.03	0.00	1	0.975
Pc1 courtship period (rainfall)	0.00	0.04	-0.12	0.01	1	0.905
Pc2 courtship period (temperature)	-0.07	0.05	-1.28	1.64	1	0.200

† Nest type 'other' were used as a reference category. Pc1: negative values: low temperatures (mean and min temperatures); low precipitation (total sum of rain and rain intensity). Pc2: negative values: low temperatures (mean, min and max temperatures) and high precipitation positive (total sum of rain and rain intensity). See full matrix of factor loadings of PCA in supplementary material S3.

Supplementary Material S8 – The relationship between breeding success and temperature and rainfall experienced during the egg-laying period expressed as **(a)** pc2lay [negative values: warm and wet weather conditions; positive values: cold and dry weather conditions]; and, **(b)** pc1lay [negative values: cold and wet weather conditions; positive values: warm and dry weather conditions]; in peregrine falcon breeding pairs across the urban gradient in Cape Town, South Africa, based on predicted values of Hurdle model (binary part). The urban gradient is represented as min (ug=1%), 1st quantile (ug=20%), median (ug=40%), 3rd quantile (ug=60%) and max (ug=80%) value: Model details in **Supplementary Material S8a.**

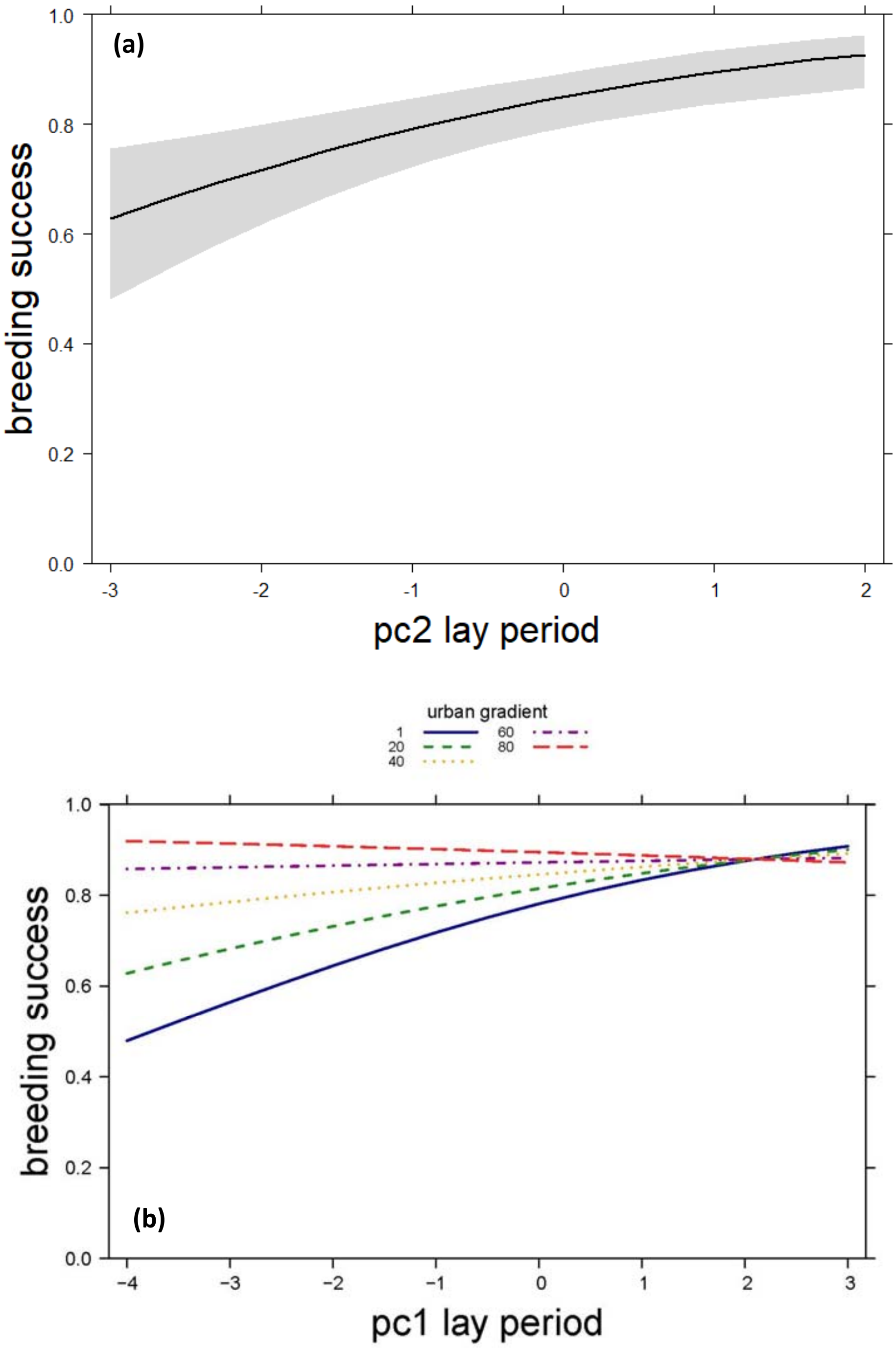

Supplementary Material S9 – The relationship between fledged brood size and temperature (pc2court) experienced during the courtship period [negative values: low temperatures (mean, min and max temperatures); positive values: high temperatures]; in peregrine falcon breeding pairs across the urban gradient (ug) in Cape Town, South Africa, based on predicted values of Hurdle model (truncated part). The urban gradient is represented as min (ug=1%), 1st quantile (ug=20%), median (ug=40%), 3rd quantile (ug=60%) and max (ug=80%) value: Model details in **Supplementary Material S8b.**

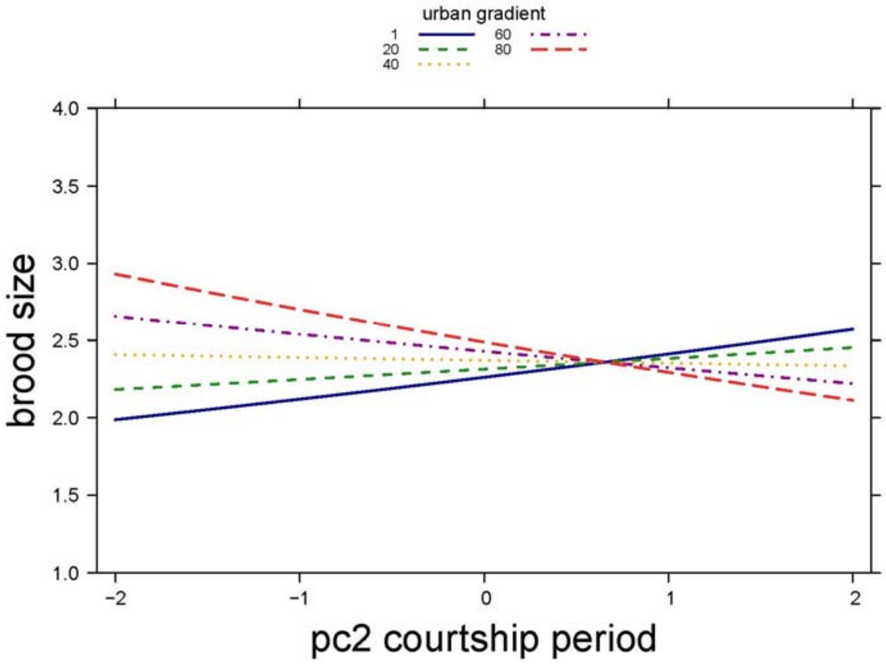

Supplement: S1 File — (PDF) [file pone.0234503.s001.pdf]
